# Comparison of African and North American velvet ant mimicry complexes: Another example of Africa as the ‘odd man out’

**DOI:** 10.1371/journal.pone.0189482

**Published:** 2018-01-03

**Authors:** Joseph S. Wilson, Aaron D. Pan, Erica S. Limb, Kevin A. Williams

**Affiliations:** 1 Department of Biology, Utah State University, Tooele, Utah, United States of America; 2 Don Harrington Discovery Center, Amarillo, Texas, United States of America; 3 Botanical Research Institute of Texas, Fort Worth, Texas, United States of America; 4 Plant Pest Diagnostics Center, California Department of Food and Agriculture, Sacramento, California, United States of America; University of Arkansas, UNITED STATES

## Abstract

Africa has the most tropical and subtropical land of any continent, yet has relatively low species richness in several taxa. This depauperate nature of the African tropical fauna and flora has led some to call Africa the “odd man out.” One exception to this pattern is velvet ants (Hymenoptera: Mutillidae), wingless wasps that are known for Müllerian mimicry. While North American velvet ants form one of the world’s largest mimicry complexes, mimicry in African species has not been investigated. Here we ask do African velvet ant Müllerian mimicry rings exist, and how do they compare to the North American complex. We then explore what factors might contribute to the differences in mimetic diversity between continents. To investigate this we compared the color patterns of 304 African velvet ant taxa using nonmetric multidimensional scaling (NMDS). We then investigated distributions of each distinct mimicry ring. Finally, we compared lizard diversity and ecoregion diversity on the two continents. We found that African female velvet ants form four Müllerian rings, which is half the number of North American rings. This lower mimetic diversity could be related to the relatively lower diversity of insectivorous lizard species or to the lower number of distinct ecoregions in Africa compared to North America.

## Introduction

Africa is geographically and biologically unique among the continents, being the only landmass currently extending across both the northern and southern hemispheric subtropical ridges, (~30°N & S) and as such, has the most extensive surface area of any continent lying within the tropical and subtropical zones [1]. While it has been observed that the tropics are generally more species rich and biologically diverse in comparison to higher latitudes (e.g., [2–3]), Africa (excluding Madagascar) has been termed the “Odd Man Out” by Richards [4] due to the depauperate nature of the African rainforest flora in terms of species richness compared to the other tropical regions. This is illustrated by the noticeable absence of or low diversity of ecologically important and widely distributed plant families (i.e. Arecaceae, Fagaceae, Lauraceae, Myrtaceae, Myristicaceae, and Orchidaceae), and the lower levels of endemic rainforest plant taxa in comparison to the other tropical regions [4]. Africa’s “Odd Man Out” status, however, extends beyond its tropical rainforest flora and is also characterized by its relatively low biodiversity and species richness in soil fungi, ants, flies, lepidopterans, aquatic amphibians, and birds in comparison to other tropical regions [4, 5–16]. Some clades, which are often associated with seasonally dry communities, are more species rich and/or diverse in Africa than on the other continental landmasses, including Acacieae and detarioid legumes, viperine snakes, bovids, termites, and velvet ants [17–23].

This last clade, the velvet ants (Mutillidae), represent a diverse group of sexually dimorphic (females are apterous), aposematic aculeate wasps that include around 4,900 named forms worldwide [19, 24–25, pers. obs.]. The African velvet ant fauna includes almost a third of the family’s species and subspecies richness (~1600 species and subspecies; [19; pers. obs.]). Recent studies have provided evidence that aposematic colored (diurnal) female velvet ants in North America represent one of the largest known Müllerian mimetic complexes in the world [26–27], yet it has not been investigated if velvet ant Müllerian mimicry complexes occur on other continental landmasses. Like those in North America, the majority of African velvet ant species also exhibit aposematic coloration. It is unclear, however, if similarly colored, unrelated African taxa have concordant geographic ranges, which would indicate mimetic rings. If multiple mimetic rings do occur in Africa, and thus form a mimicry complex, it would be expected that the number of mimetic rings making up this complex would be similar to, or more phenotypically diverse compared to those found in North America, given the greater geographic area and higher velvet ant species richness in Africa. Alternatively, the generally depauperate nature of the African fauna and flora could be reflected in a less diverse African mimetic complex than has been reported in North America [26–27].

We hypothesize that female Müllerian mimicry ring diversity is a reflection of predator diversity/heterogeneity and ecological heterogeneity (which may not be mutually exclusive) rather than simply an echo of species richness. In this regard, we predict that a diverse aposematic clade, like the African velvet ants, while species rich and diverse, will have a less diverse mimicry complex compared to mimicry complexes from areas with a more diverse/heterogeneous predator fauna and/or a higher number of distinct ecological communities.

To test this hypothesis, we assessed the color pattern and distributions of 304 diurnal velvet ant taxa (58 genera from 3 subfamilies), which represents approximately 30% of the described diurnal female species (1010 species and subspecies) known from the African continent (excluding Madagascar). We ask (1) do African female velvet ant Müllerian mimicry rings exist, and if so, are the discrete mimetic rings geographically restricted, thus representing a continental mimicry complex, (2) are the number of African mimetic rings comparable to the number of rings in the North American mimicry complex, (3) is the familial/generic diversity and species richness of insectivorous/omnivorous squamates, particularly iguanians, between both continental faunas related to the mimetic diversity of velvet ants, and (4) is the number of terrestrial ecoregions and landmass sizes of both continents related to the mimetic diversity of velvet ants?

## Methods

### Specimen sampling

Female African velvet ant specimens (261 taxa: S1 Fig) were photographed from entomological loaned specimens from the following institutional collections: American Museum of Natural History, California Academy of Sciences, Carnegie Museum of Natural History, Florida Department of Agriculture, Lund University, Muséum National d’Histoire Naturelle, National Museum of Natural History, Naturalis Biodiversity Center, Natural History Museum–London, Royal Belgian Institute of Natural Sciences, Texas A&M University, University of California Davis, University of California Riverside, University of Minnesota Saint Paul, and Utah State University. Specimens were photographed in dorsal and lateral views by one of the authors (K.A. Williams). Forty-three additional taxa were included in the morphological analysis based on published descriptions and associated illustrations. Moreover, an additional 48 diurnal female velvet ant taxa that are known to occur in the Palearctic zone of Africa (from the South Saharan Steppe and Woodlands terrestrial ecoregion northward) were examined; these collection specimens, however, were from localities outside of Africa (Mediterranean Europe and the Middle East). Although not used in the analysis, these 48 specimens were compared with the derived mimicry ring distributions to determine if the study’s results were supported by these additional taxa [28].

### Morphological analysis

Morphological analyses were performed to determine if the velvet ant species occurred in discrete groupings (indicative of mimicry rings). Methodology and characteristics analyzed follow Wilson et al. [26], including coding of head primary (background) and secondary colors, mesosoma background and secondary colors, petiole color, the presence or absence of a contrasting setal spot on the petiole, metasoma background and secondary colors, the presence of a third color/maculae on the second tergite of the metasoma (T2), contrasting dark and light pattern on T2, light setal fringes on the apical tergites (T3 –T5), integument color, leg color, and setal length (S1 Table). Velvet ant species were grouped into putative mimicry rings based on visual similarities. These *a priori* groupings were then examined using nonmetric multidimensional scaling (NMDS) and a permutational analysis of variance (PERMANOVA) calculated using the isoMDS function in the MASS package and the adonis function in the vegan package in R (R Foundation for Statistical Computing). A Gower distance matrix was utilized for the NMDS, due to analysis of categorical data [26–27, 29].

### Distributional analysis

Distributions of the velvet ant species were derived from specimen label locality data and published distributions and localities [28, 30–57]. These localities were used to determine the terrestrial ecoregion distributions of each taxon using the World Wildlife Foundation’s (WWF) terrestrial ecoregion system. The species distributions were overlaid on each other in Adobe Photoshop CS6 to derive the distribution of each species and their association with possible mimicry rings derived from the NMDS analysis.

### Analysis of predator richness

Information on lizard (including amphisbaenians) familial and generic diversity and species richness for Africa (excluding Madasgascar, Comoros, Seychelles, and Mascarenes) and North America (including the Greater and Lesser Antilles) was obtained from publications and reptile databases [58–65]. These measures of diversity were then compared between Africa and North America.

### Analysis of ecoregion diversity

To compare ecological community diversity and heterogeneity between Africa (excluding Madagascar, Comoros, Seychelles, and Mascarenes) and North America (including Central America and the Caribbean–Bahamas, Greater Antilles, and Lesser Antilles), we compared the number of WWF terrestrial ecoregions of the two areas, excluding tundra biome communities because no velvet ant or lizard species occur there [66–70]. This system was used because it provides a global classification system for ecological communities on finer scales than previous biogeographic systems and incorporates endemic taxa and ecological processes and phenomena [68].

## Results

### Mimicry results

**Fig 1:**
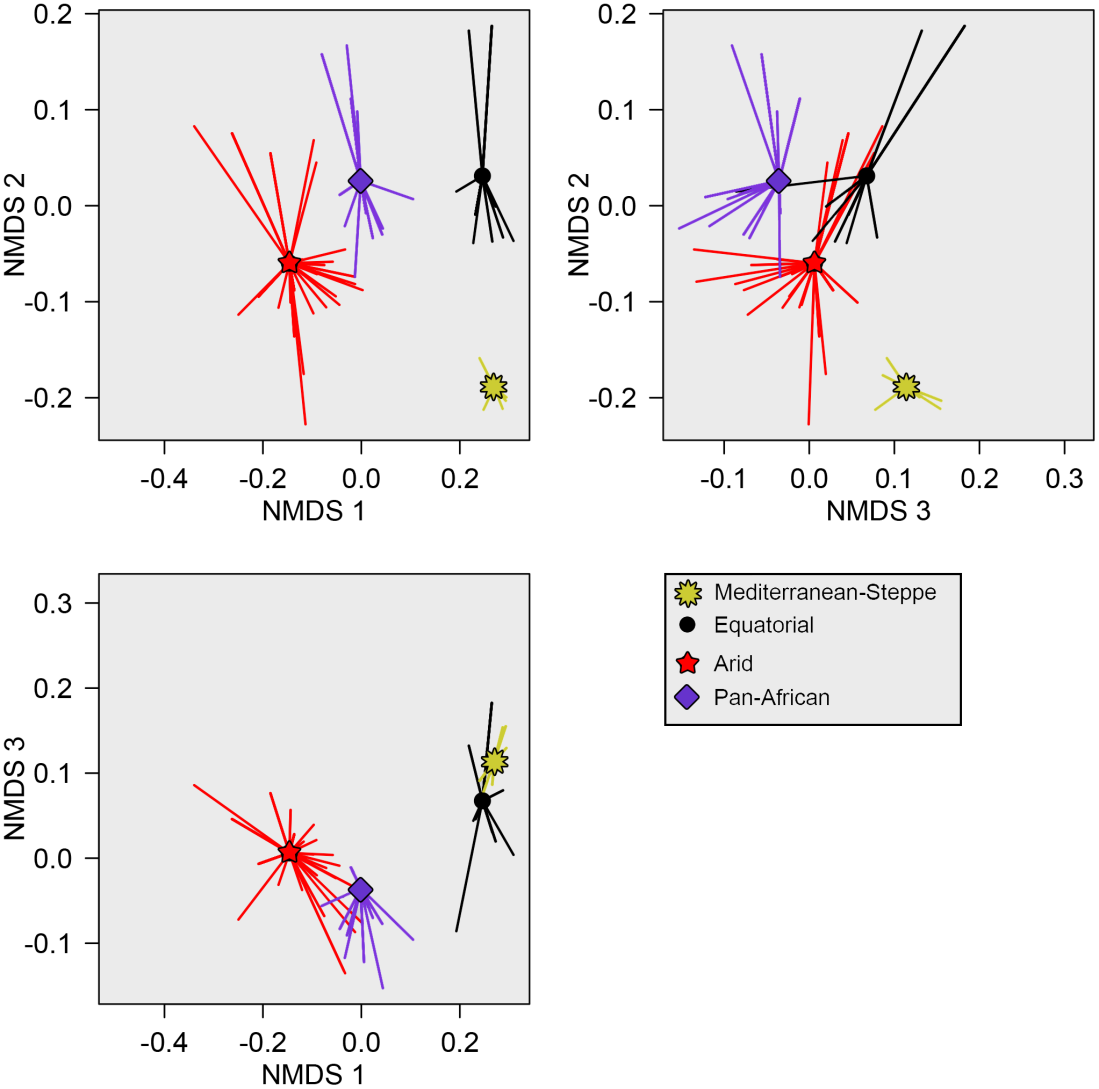
Velvet ant mimicry complex in 3D ordination space. Velvet ant mimicry complexes are differentiated in ordinal space (NMDS). In each comparison of the three NMDS axes, the mean values for each mimicry ring are denoted by symbols, with lines drawn from the means to individual species values.

**Fig 2:**
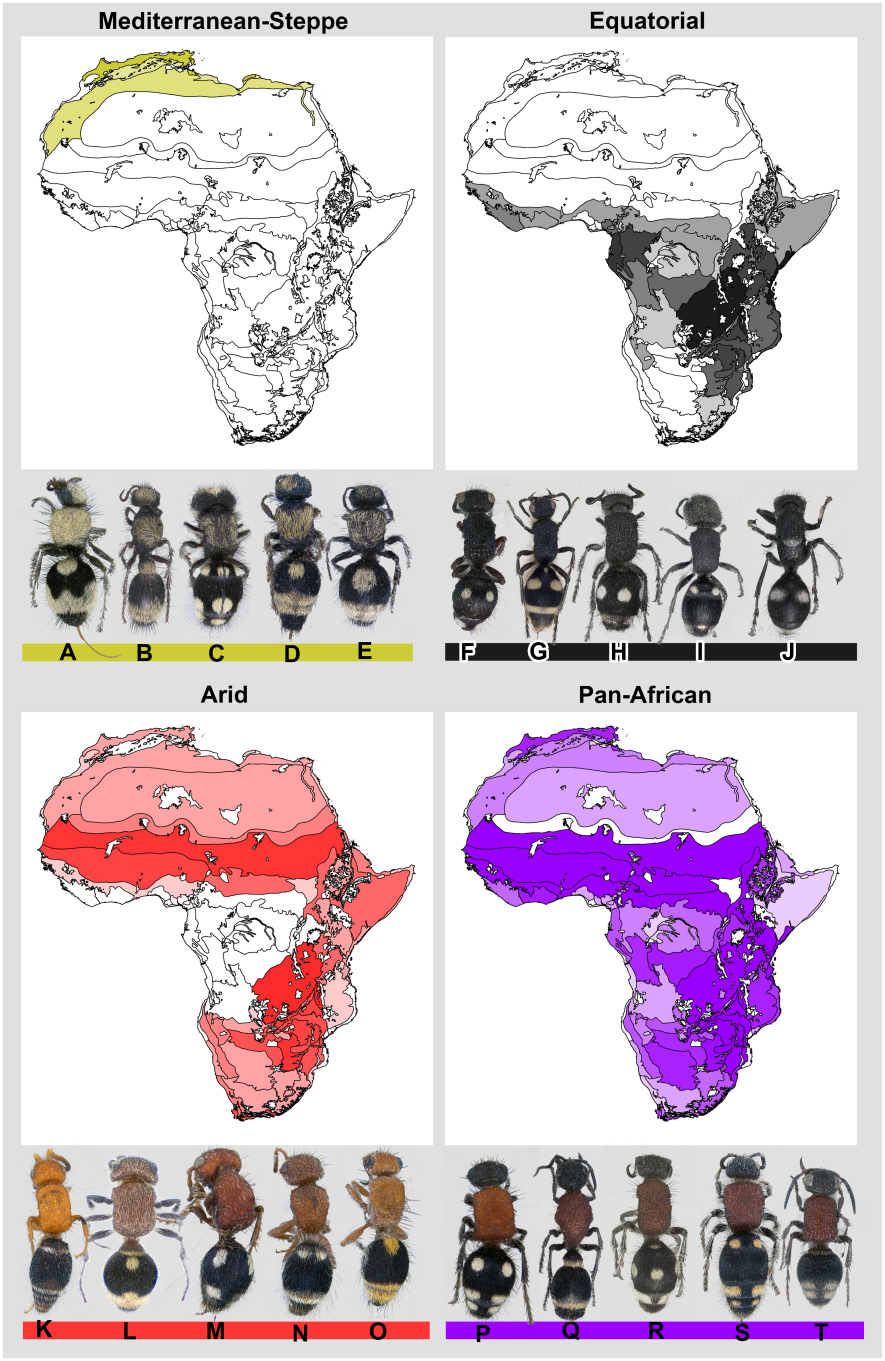
African velvet ant mimicry rings. The morphological and geographic ranges of the four African velvet ant mimicry rings. Each mimicry ring is represented here by five species. These species were selected because they are morphologically closest to the estimated mean for each mimicry ring (see Fig 1). The geographic range of each mimicry ring is presented based on distributional analyses that examined the known range of each species involved in each mimicry ring. The species pictured here are as follows: A *Dasylabris arabica*, B *Stenomutilla argentata*, C *Ronisia maculosa*, D *Nemka viduata tunensis*, E *Smicromyrme mareotica*, F *Carinotilla* cf *stipnopyga*, G *Trispilotilla africana*, H *Smicromyrme tettensis melanothoracica*, I *Mutilla astarte ignava*, J *Dolichomutilla scutellata*, K *Tropidotilla fimbriata*, L *Sulcotilla sulcata*, M *Glossotilla mogadiscioana*, N *Dasylabroides latona ruficeps*, O *Dasylabris bassutorum*, P *Trogaspidia sulcicada*, Q *Stenomutilla analis*, R *Smicromyrme tettensis tettensis*, S *Odontomutilla calida calida*, T *Cephalotilla ceratophora*.

Based on our analyses, four distinct mimicry rings can be found in Africa (Fig 1) (each of the four mimicry rings is morphologically distinct: the overall effect of mimicry ring as a categorical variable was F_4,271_ = 215.55, R^2^ = 0.761, P < 0.001; and can be distinguished based on integument and setal coloring and patterns (Fig 2). This indicates that a continental mimicry complex does exist for Africa and is made up of four mimicry rings; the Mediterranean-Steppe mimicry ring, the Equatorial mimicry ring, the Arid mimicry ring, and the Pan-African mimicry ring.

The Mediterranean-Steppe mimicry ring is associated with the Mediterranean mountains, highlands, coastal desert, and steppe areas of northernmost Africa, with the core centered in the Atlas and Aurés Mountains. This mimicry ring does not appear to extend southward into the core of the Sahara Desert itself, but occurs along its fringe in the steppe, woodland, and Nilotic areas. The Mediterranean- Steppe ring also appears to be absent from the highlands in the Saharan interior (Hoggar Mountains, Tibesti Mountains, Tassili n’Ajjer, and Aïr Massif; [71]). Members within the Mediterranean-Steppe mimicry ring have black integumental coloration with bronze, gold, or white setal coloration on the head, mesosoma, and metasoma. The metasoma typically has a bronze, gold, or white colored band on the apical tergites.

The Equatorial mimicry ring is centered in the equatorial region and is found on both the western and eastern sides of the East African Rift. With two main cores, one extending from the Horn of Africa to northeastern South Africa, especially associated with coastal and montane forests, miombo, bushland thickets, and bushveld in the east and southeast, and a western core associated with the moist and wet forest communities of the Guineo-Congolian region. Dolfuss [72] and Nonveiller [51] both noted the association of velvet ant taxa with black mesosomae (‘melanic/melanistic forms’) in forested communities compared to closely related taxa with red mesosomae in savannas. This mimicry ring is absent from the Cape, Karoo, and most of the Kalahari areas of southwestern Africa. Members of the Equatorial mimicry ring possess black integument with sparse or no setae on the mesosoma, and often with white, yellow, or orange macula(e) on the first and/or second tergite(s) and white or light colored setal bands (sometimes segmented) on the apical tergites.

The Arid mimicry ring is distributed in the semi-arid and arid regions of the continent, particularly areas that have extensive dry seasons. This ring is absent from the more mesic portions of the continent, the Guineo-Congolian region, including the Congo Basin and the West and Central African rain forests. The Arid mimicry ring is particularly abundant in the Sahelian, Sudanian, and Somalian portions of northern and northeastern Africa, occurs in the Sahara Desert and associated steppe communities, East Africa’s Great Rift Valley, and the Zambezian, Kalahari, Namib, and Eastern Cape regions of southern Africa. The Arid ring is characterized by red coloration (either due to integument or setae) on the head and mesosoma. The metasoma is black or dark in coloration and often has white, yellow, orange, or gold colored maculae. A number of members of this ring that are distributed in southern Africa from the miombo communities of south-Central Africa and extending through the Natal, Kalahari, Karoo, Namib, and Cape regions lack maculae on the second metasomal tergite.

The Pan-African mimicry ring is distributed throughout Africa and appears to be associated with multiple ecological communities and climate regimes, being found in arid and mesic environments. This mimicry ring is by far the most speciose and diverse with 187 of the 304 taxa examined included in this ring. The Pan-African mimicry ring consists of individuals with black/dark colored head, red mesosoma, and black/dark colored metasoma often with lines and/or maculae of white, yellow, gold, or orange colored setae.

While we were not able to examine every named species from Africa, our sample does contain a representative sample of African velvet ants. For example, for the species rich genus *Trogaspidia*, our study included 22 described species and seven unidentifiable morphospecies. Bischoff’s [33] treatment of the genus included 115 described species and subspecies of females that are currently assigned to *Trogaspidia*, [these belong to his *T*. *divisa* (93 named forms) and *trigonophora* (22 named forms) species-groups]. Based on his identification keys, these species can be tentatively assigned to the following mimicry rings: 15 Equatorial forms (13%), 12 Arid forms (10%), and 88 Pan-African forms (77%). Of the 29 *Trogaspidia* photographed and analyzed in our study, three were Equatorial forms (10%), four were Arid forms (14%), and 23 were Pan-African forms (76%). Although in the present study we were only able to directly examine fewer than 30% of the recognized *Trogaspidia* female forms, we ended up with similar ratios for each mimicry ring. In addition, the genus is not known to include species that do not conform to one of the mimicry rings described herein.

### Predator richness results

African non-ophidian squamate (lizard and amphisbaenian) familial diversity is only about 65% compared to that of North American (including Central America and Caribbean) fauna (15 versus 23 families; [58–63, 65]. Generic lizard diversity, however, is more substantial in Africa than North America with 118 and 66 genera, respectively [58–63, 65]. In terms of species richness, both continents are comparable with North America having a higher, but relatively comparable, lizard species richness in comparison to Africa, approximately 980 and 940 species, respectively [58–63, 65]. Insectivorous iguanian lizard familial and generic diversity and species richness is higher in North America (7 families, 19 genera, and over 500 species) compared to Africa (2 families, 12 genera, and over 175 species; [58–63, 65]. Lizard species richness in Africa is highest in the Central Zambezian Miombo Woodlands, Drakensberg Montane Grasslands, Woodlands, & Forests, Nama Karoo, Namibian Savanna Woodlands, Northern Zanzibar-Inhambane Coastal Forest Mosaic, Somali Acacia-Commiphora Bushlands & Thickets, Succulent Karoo, and Zambezian and Mopane Woodlands ecoregions [62, 69]. In North America the ecoregions with the highest species richness of lizards include the California Coastal Sage & Chaparral, Central American Dry Forests; Central American Pine-Oak Forests, Chiapas Depression Dry Forests, Chihuahuan Desert, Isthmian-Atlantic Moist Forests, Petén-Veracruz Moist Forests, Sierra Madre Occidental Pine-Oak Forests, Sonoran Desert, and Southern Pacific Dry Forests [58–65, 67].

### Ecoregion diversity results

The African continent and associated small islands (São Tomé, Príncipe, Annobon, Bioko Island, Canary Islands, Cape Verde Islands, and Zanzibar) includes 108 terrestrial ecoregions within 9 biomes extending over nearly 29,200,000 km^2^ [69]. Africa lacks four distinct biome types: boreal forest/taiga, temperate broadleaf and mixed forests, temperate grasslands and savanna, and tropical coniferous forests [69]. North America, including Central America and Caribbean Islands, contains 168 terrestrial ecoregions within 13 biomes and extends over 17,521,000 km^2^ (excluding tundra biome and associated ecoregions; [66–67, 70]. Some biomes, like the Boreal Forest/Taiga, likely house few, if any, velvet ant species and if these are also excluded, North America still consists of 151 terrestrial ecoregions [66–67].

## Discussion

Our results clearly show that Africa does indeed harbor a large velvet ant mimicry complex, composed of four distinct mimicry rings. What is particularly intriguing, however, is that this African velvet ant mimicry complex is less phenotypically diverse than the North American mimicry complex (i.e., there are only 4 African rings compared to 8 North American rings) [26–27]. This lack of phenotypic diversity is especially interesting given the fact that the African velvet ant fauna is more species rich than North America and that Africa is over 1.35 times larger than North America [67, 69, 73]. So, even while velvet ants are more species rich in Africa than in North America, the “odd man out” observation of low richness in African taxa holds true if mimetic diversity is taken into account. There are several potential explanations for this lack of phenotypic diversity in the African velvet ant mimicry complex that we will discuss below.

First, the apparent lack of mimicry ring diversity in the African velvet ant mimetic complex could be related to the diversity of predators. It has been suggested that insectivorous/omnivorous squamates, particularly iguanians, might be a major predator of velvet ants and that they likely play a role in the development of aposematic coloration [29]. Furthermore, in North America there is a link between the species richness of lizard predators and the richness of mimicry rings [29]. While the lizard species richness between Africa and North America is comparable (~940 and 980 species respectively), Africa has a much lower lizard heterogeneity per km^2^ due to its much greater landmass size, especially when considering that large portions of North America are covered by tundra and boreal forest and taiga that include essentially no species of lizards or velvet ants [62, 64, 67]. Furthermore, lizard species richness is spread across the African continent, with many ecoregions harboring relatively rich lizard communities [62]. Alternatively, North American lizard communities are much less equally distributed, with the majority of lizard species being found in only a handful of ecoregions (primarily those in the arid southwest and in the Neotropics [74]. This concentration of lizard richness in North America could have increased selective pressure on the evolution of aposematism and driven the development of a more phenotypically diverse velvet ant mimicry complex compared to the more dispersed lizard richness (and lower phenotypically diverse velvet ant mimicry complex) found in Africa. This is supported by the concordance of the patterns of lizard richness and velvet ant mimicry rings on both continents. For example, the Central Zamebezian Miombo Woodlands is among the richest ecoregions for velvet ants, both in terms of species richness and mimicry ring richness (three of four mimicry rings). This same ecoregion also houses one of the richest lizard communities in Africa [62]. Similarly, in North America, the Sonoran and Chihuahuan deserts include five of the eight North American velvet ant mimicry rings [75] and are home to the some of the richest lizard communities in North America [74].

These patterns of lizard diversity, and the associated patterns of velvet ant diversity, are likely also connected to ecoregion diversity. Africa, while larger in size than North America, contains fewer ecoregions (108 total ecoregions in Africa, 168 in North America). Furthermore, many of Africa’s ecoregions are much larger than those found in North America. For example, Africa has seven ecoregions that are over 1 million km^2^ in size. North America’s largest ecoregions are all smaller than 1 million km^2^ and most of the largest are in Boreal Forest/Taiga Biomes. This means that much of the North American habitat conducive to velvet ants (and lizards) is condensed and highly variable with many ecoregions found in relatively small areas. Africa, nearly in its entirety, provides suitable velvet ant (and lizard) habitat, with broad swaths of land maintaining similar habitat characteristics. That habitat variability in North America compared to Africa could be associated with the difference in mimetic diversity between the two continents. Habitat variability could lead to diversification in mimicry complexes; as climatic shifts drove different communities into isolated areas (particularly during the Pleistocene), different selective pressures or genetic drift could have resulted in phenotypically distinct mimicry rings. The more heterogeneous a landmass is, the more likely ancestral communities would have shifted into multiple distinct habitats, resulting in more unique mimicry rings.

It is clear that Africa houses a less phenotypically diverse mimicry complex than North America. Despite this lower diversity, some aspects of the African complex should be discussed further. The Mediterranean-Steppe ring, for example, is the most geographically restricted ring on the African continent and is associated with the Palearctic portion of the continent [68–69]. The ring also extends into the Iberian Peninsula and, as a whole, the African and European portion of the ring likely represents the western extension of a Müllerian mimicry ring that also extends into the steppe areas of the Near East, around the Caspian Sea and into the highland areas of Central Asia in the east. The Mediterranean-Steppe mimicry ring is also the smallest of the four African mimicry rings in terms of species richness. It is notable that the whitish or bronze setae coloration of these mainly highland taxa are reminiscent of similar setal coloration found in the Madrean or Desert Mimicry rings of North America, which are also generally associated with upland environments [26–27]. The restricted nature of the Mediterranean-Steppe ring and the few species that contribute to it, suggest that this ring only recently evolved on the African continent. The northwestern portion of Africa has a noticeably high density of lizard taxa, particularly agamids and lacertids (i.e. *Acanthodactylus* and *Mesalina*) that are known to take ants as a high proportion of their diet [62, 76–78]; these are likely predators of the Mediterranean-Steppe ring.

The Equatorial ring is interesting in that it can be found in both mesic and xeric ecological communities, but is particularly predominant in the tropical moist/wet forests of the Guinean-Congolian region, the Eastern Arc, East African Montane, and coastal forests, and miombo communities of southeastern and East Africa. The black head and mesosoma is not necessarily indicative of aposematic coloration. A number of Afrotropical forest ants (ex. *Ankylomyrma*, *Aphomomyrmex*, *Paltothyreus*, *Phasmomyrmex*, & *Psalidomyrmex*) also have entirely black integument, suggesting a cryptic adaptation [79]. The bright colored maculae and/or setal bands on the tergites of Equatorial mimicry ring velvet ants may provide an aposematic warning to possible predators and discriminates these female velvet ants (mutillids) from rainforest ants (formicids). This ring also appears to be the only one that is a definitively endemic mimicry ring in Africa (although it should be noted that similar colored taxa can be found in Madagascar as well as in Australian and South American taxa).

Both the Arid mimicry ring and the Pan-African mimicry ring represent color patterns that are found in velvet ants across the globe. For example, in North America the Black-headed Timulla and Red-headed Timulla rings share these color patterns [27]. Due to the widespread occurrence of this coloration, it is possible that it represents an ancestral pattern for velvet ants. Another interesting similarity between mimicry complexes on the two continents is that those mimicry rings characterized by having predominantly black coloration with starkly contrasting abdominal patterns are both found primarily in equatorial areas. The Tropical mimicry ring (North America) and the Equatorial mimicry ring (Africa) are both restricted to the tropics (areas between 23.5 degrees N and S). Some of the most widespread mimicry rings in North America (western, Desert, and Eastern rings) do not have comparable African counterparts in terms of coloration. This could be related to the fact that these unique North American rings are found primarily in the Nearctic regions of the continent, while Africa does not have comparable temperate grassland biomes.

Furthermore, it is apparent in many of the African and North American mimicry rings that the abdominal color patterns are highly variable, even within a given ring. For example, some members of the Pan-African ring have nearly completely black abdomens, while others have large patches of white setae, and these setal patterns often differ between species and regions. Similar patterns of variability can be found in the Equatorial, and Arid African rings, as well as in the Tropical, Madrean, and red-headed and black-headed Timulla rings in North America. This variability might indicate that with more sampling at finer scales, these large mimicry rings might be subdivided into smaller more specific sub-rings.

It might be asked why velvet ants have been so successful as a Müllerian mimicry complex. We suggest that the diversity and success of velvet ant mimicry complexes likely is related to the following series of observations and hypotheses [29]. First, ants (Formicidae) are one of the most prolific and ecologically dominant organisms in tropical and subtropical environments [79–80]. Because of the wingless nature of female velvet ants, they resemble ants, hence the common name ‘velvet ant.’ Even more than true ants, velvet ants are highly defended with painful stings, extremely hard cuticles, pungent chemical secretions, and stridulation warning sounds [26]. So, because of their similarity to ants, predators that often eat ants could pose a threat to velvet ants. We suggest that the evolution of aposematism, including the long setae that many velvet ants exhibit, likely evolved as a way to differentiate themselves from true ants as a way to protect themselves from ant specialist predators. Most Diurnal velvet ants either have bright contrasting patterns, which are not seen in true ants, or they have long setae, also rarely seen in true ants. Many velvet ant species have both long setae and aposematic color patterns.

## Conclusion

Our study of mimicry in African velvet ants clearly shows that African velvet ants do participate in a large Müllerian mimicry complex. Furthermore we find that, while four distinct mimicry rings are found in Africa, this is less than the number of mimicry rings found in North America, further illustrating the “odd man out” concept of African species richness (in this case mimetic richness). We suggest that the lower number of African mimicry rings could be related to the diversity and distributions of lizards, with Africa having more uniform distribution of lizard species compared to North Americas lizard diversity that is concentrated in a few ecoregions. Finally we suggest that the lower number of African mimicry rings is likely associated with the lower number of ecoregions in Africa compared to North America.

These findings of species rich mimicry rings in both Africa and North America expand our understanding of mimicry in velvet ants and clearly show that velvet ants form the worlds largest known mimicry complex with nearly 600 species involved in 12 mimicry rings (~350 species in eight mimicry rings in N. America and at least 250 species in four rings in Africa).

## Supporting information

S1 Fig African velvet ant mimicry rings. Images of all of the velvet ant species included in the analysis organized into their respective mimicry rings.
(PDF)

S1 Table African velvet ant morphological characters. Characters and character states for each of the velvet ant species included in the NMDS analysis.
(XLSX)
