# Supplementary material for: Comparison of African and North American velvet ant mimicry complexes: Another example of Africa as the ‘odd man out’
Source: PLoS One. 2018 Jan 3;13(1):e0189482. doi: 10.1371/journal.pone.0189482 (PMC5752001; doi:10.1371/journal.pone.0189482)
Supplement: S1 Fig — Images of all of the velvet ant species included in the analysis organized into their respective mimicry rings. (PDF) [file pone.0189482.s001.pdf]

ARID RING

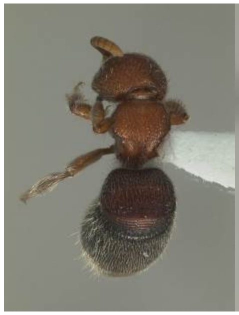

***Bischoffiella* sp.**

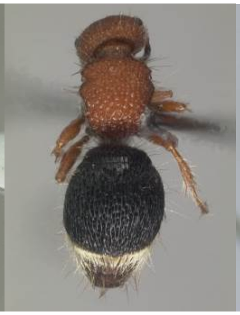

Brachymutilla scabrosa

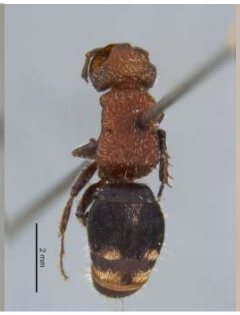

Cataractaetilla angolana

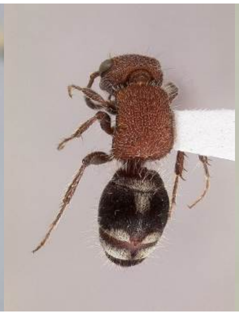

Cataractaetilla cataractae

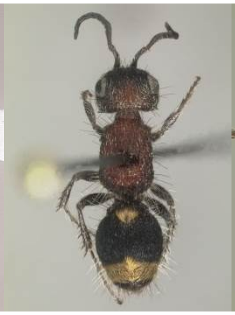

Cephalotilla dichromatica auratissima

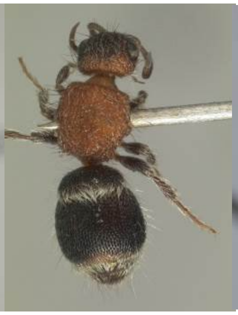

Chrestomutilla glossinae

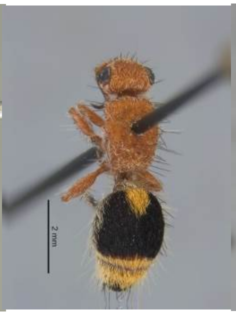

Dasylabris bassutorum

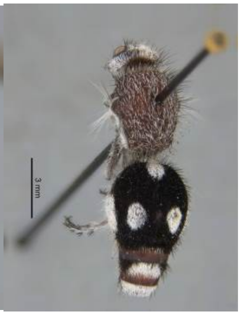

Dasylabris deckeni argenticeps

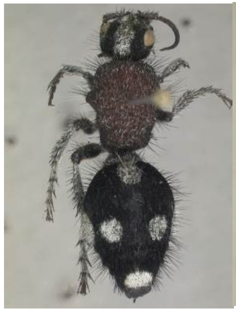

Dasylabris deckeni signaticeps

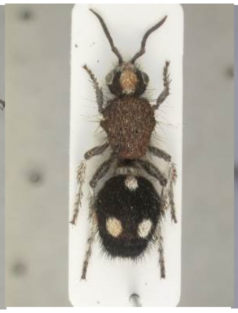

Dasylabris dora

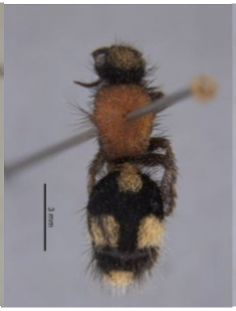

Dasylabris lybica

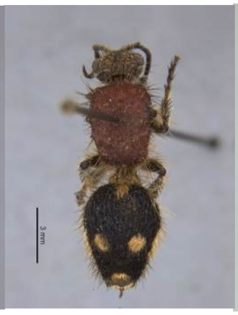

Dasylabris mephitidiformis

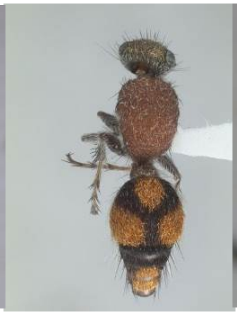

Dasylabris merope argyria

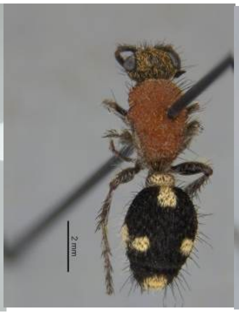

Dasylabris neavei neavei

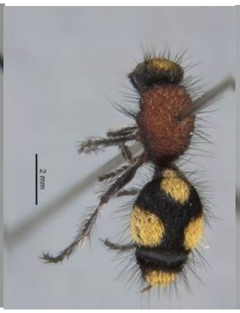

Dasylabris neavei semiaurata

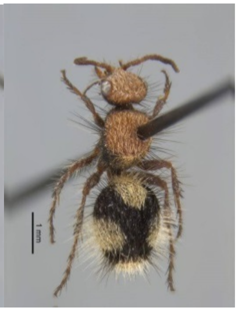

Dasylabris rufocephala

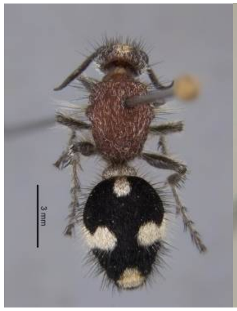

Dasylabris togoana

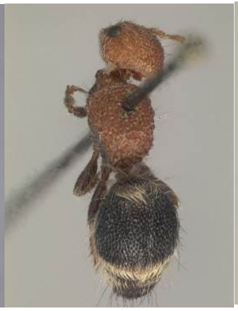

Dasylabroides caffra caffra

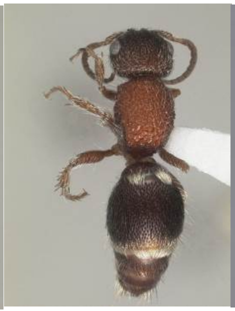

Dasylabroides foveilabris

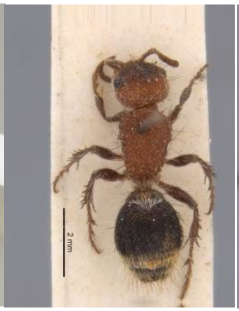

Dasylabroides philyra

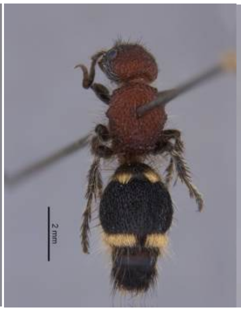

Dasylabroides* cf. *alluaudi

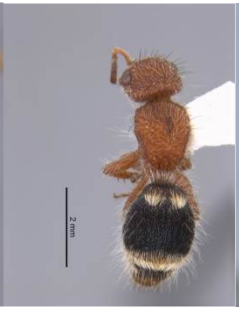

Dasylabroides latona ruficeps

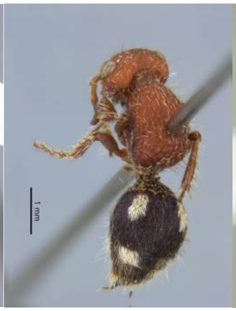

Glossotilla mogadiscioana

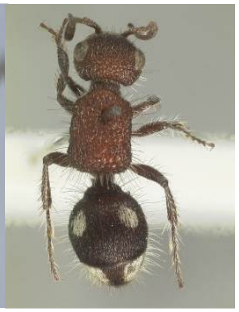

Glossotilla shiratiensis

ARID RING

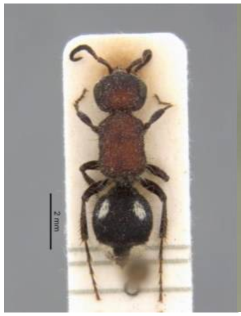
Glossotilla suavis abessinica

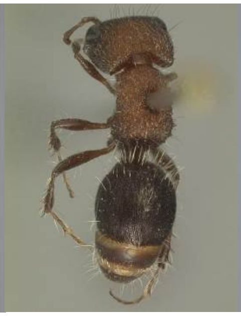
Labidomilla edentata

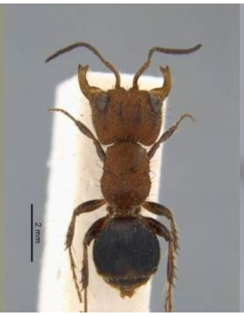
Labidomilla tauriceps

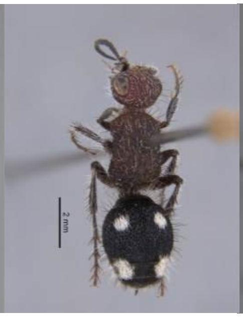
Mimecotilla djibutina rubriceps

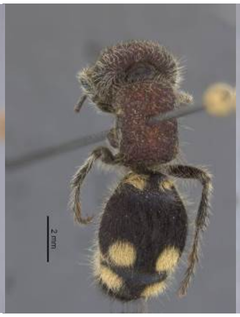
Mutilla mniszechi mniszechi

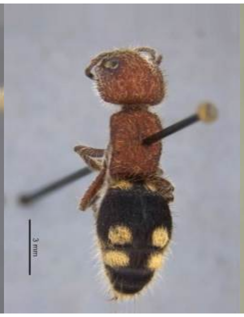
Mutilla mniszechi senegalensis

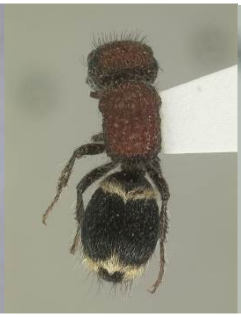
Mutilla scabrofoveolata

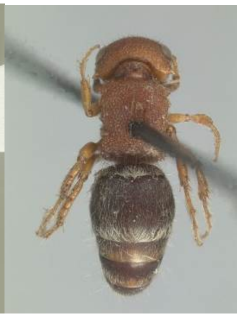
Myrmilla s.l. sp. nov.

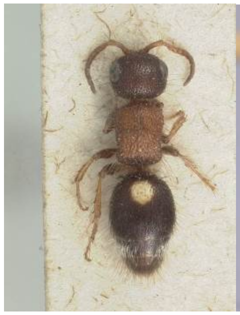
Myrmotilla lucasiella

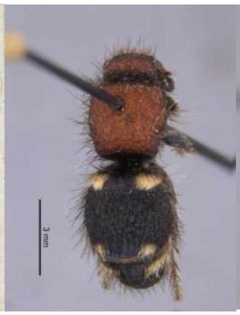
Odontomutilla ovata

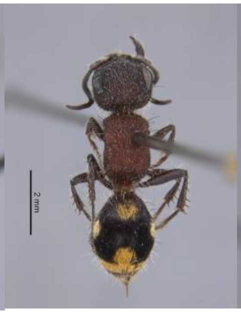
Odontotilloides quadrimaculata

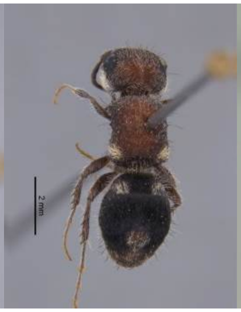
Omotilla conjunctoides

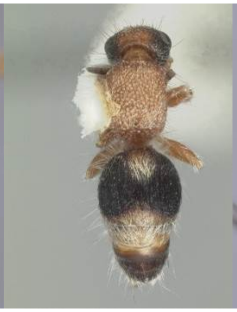
Physetopoda gridellii

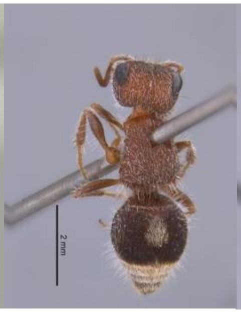
Pygomilla sp.

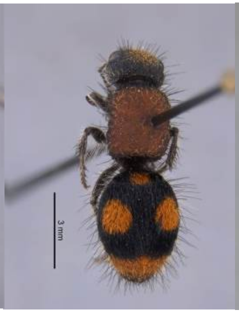
Ronisia andromeda ansifera

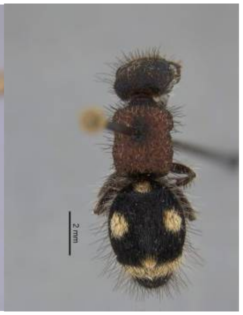
Ronisia andromeda arsinoe

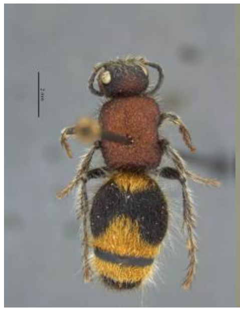
Ronisia electra

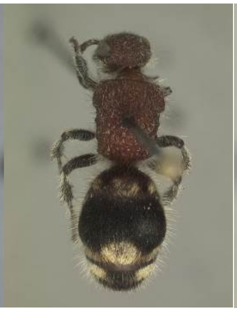
Ronisia idonaeformis

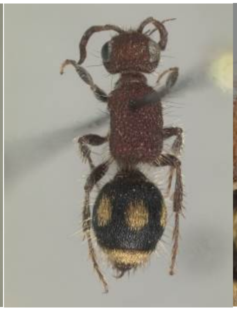
Smicromyrme longigena

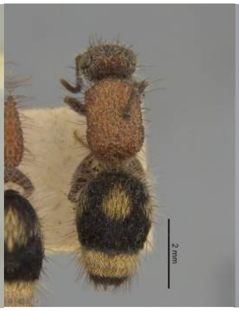
Smicromyrme melpomene hecuba

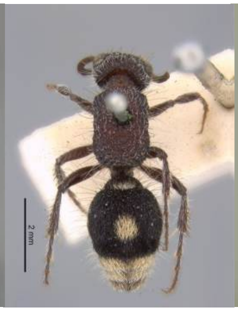
Smicromyrme pruinosa

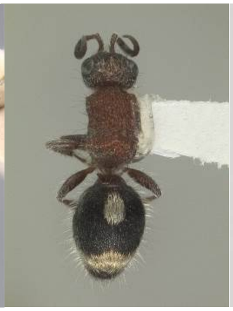
Smicromyrme sp.

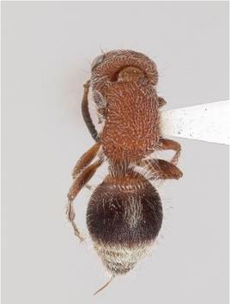
Strangulotilla bechuana

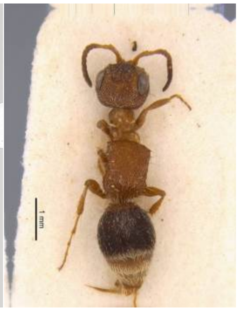
Strangulotilla pseudominuta

ARID RING

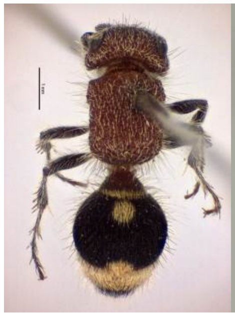
Sulcotilla sulcata

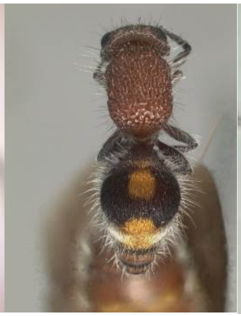
Sulcotilla* cf. *sulcata

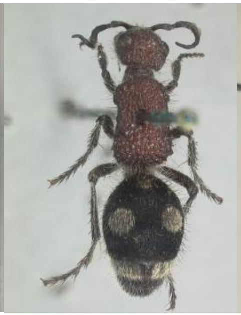
Trispilotilla senegalensis

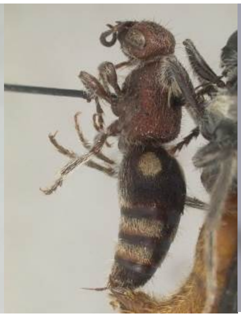
Trogaspidia floralis

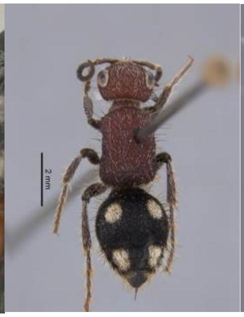
Trogaspidia samburuana

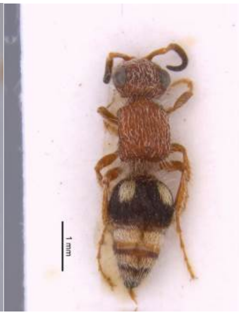
***Trogaspidia* sp.**

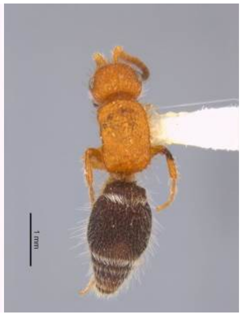
Tropidotilla fimbriata

Other Arid Ring species not pictured:

Apteromutilla aethra
Apteromutilla pandora
Hadrotilla helle
Smicromyrmilla arnoldi
Smicromyrmilla dentifera

COSMOPOLITAN RING

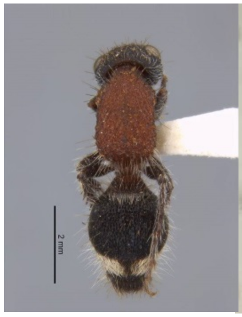

Arcuatotilla arcuaticeps

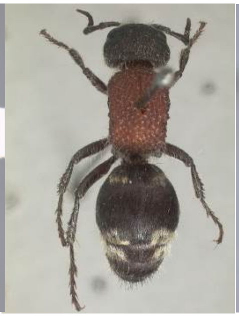

Barymutilla pythia dasya

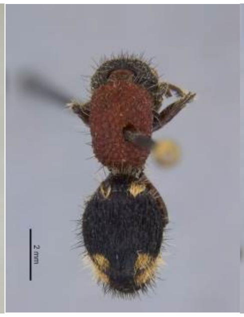

Barymutilla pythia pythia

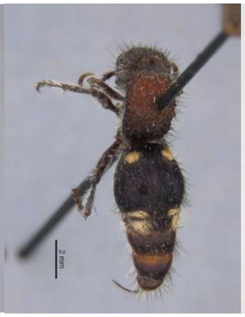

Barymutilla pythia radovae

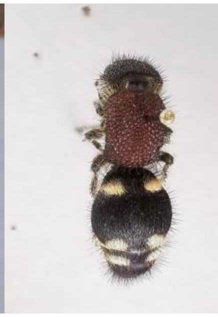

Barymutilla sp.

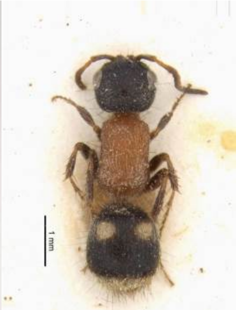

Bidecoloratilla negrei

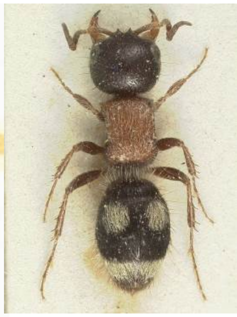

Blakeius bipunctatus

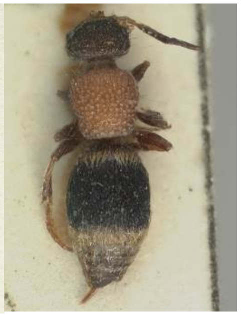

Brachymutilla gynandromorpha

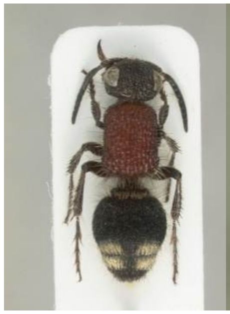

Cephalotilla ceratophora

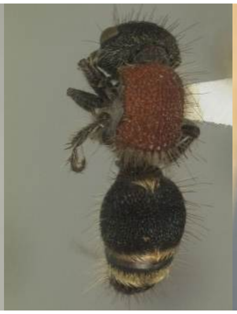

Cephalotilla denticeps

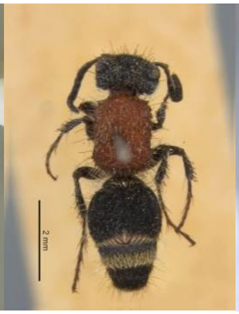

Cephalotilla granulostriata

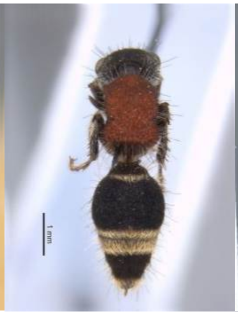

Cephalotilla obscurior

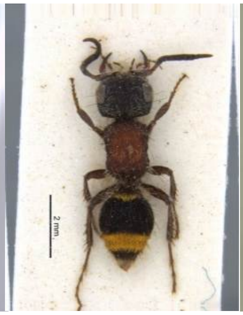

Cephalotilla pulchra

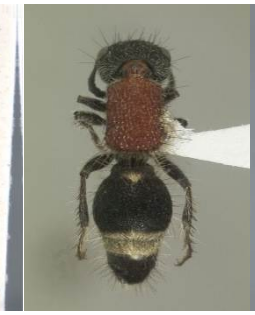

Cephalotilla sabargumae

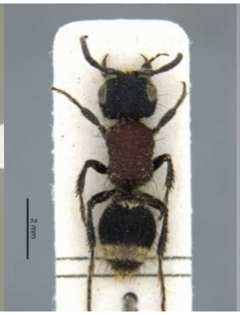

Cephalotilla sabargumaeformis

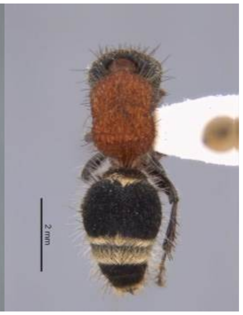

Cephalotilla sinuosiceps

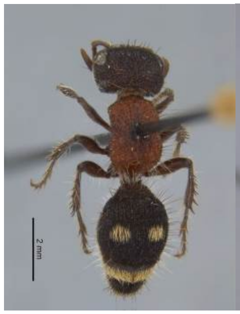

Ceratotilla dolosa ino

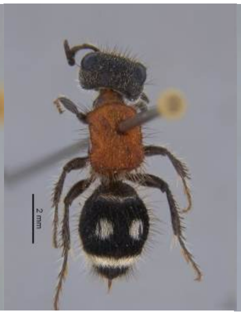

Ceratotilla dolosa septemmaculata

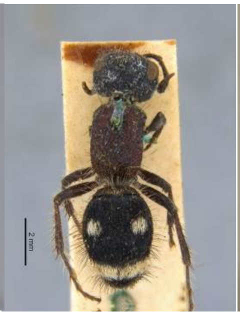

Ceratotilla cf. *obtusata*

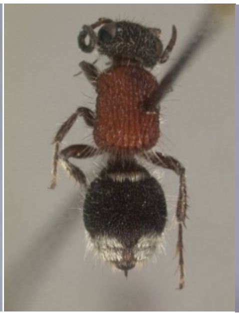

Chaetomutilla fornasinii

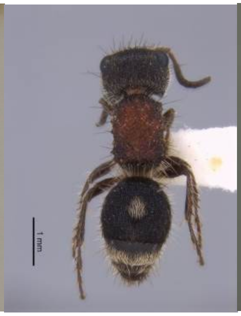

Clinotilla sp.

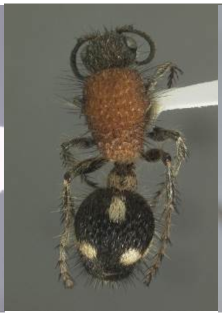

Dasylabris inflata subcarinata

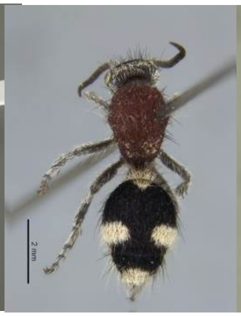

Dasylabris kameruna

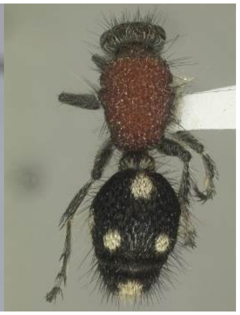

Dasylabris mephitis

COSMOPOLITAN RING

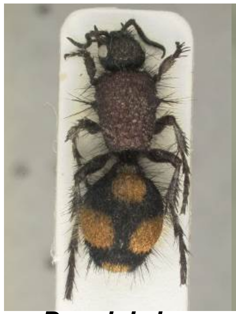
Dasylabris merope merope

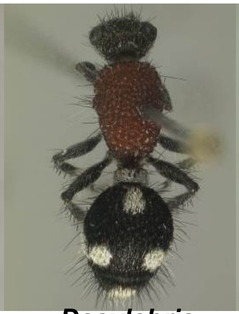
Dasylabris stimulatrix affinissima

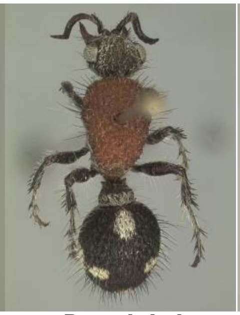
Dasylabris stimulatrix stimulatrix

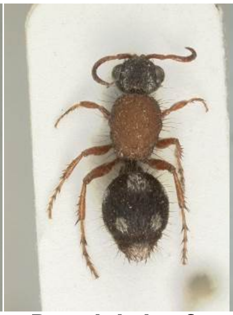
Dasylabris cf. *permaculata*

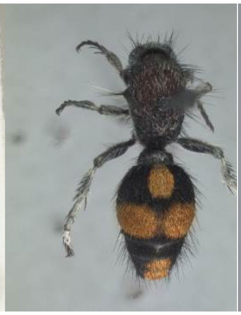
Dasylabris cf. *porphyrea*

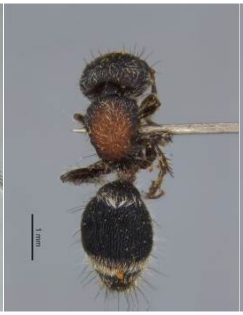
Dasylabroides alcis

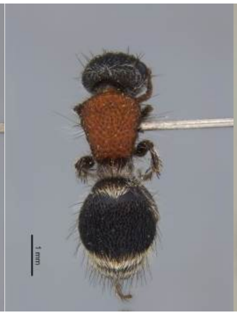
Dasylabroides caffra nigriceps

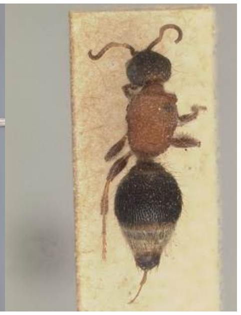
Dasylabroides eumenes

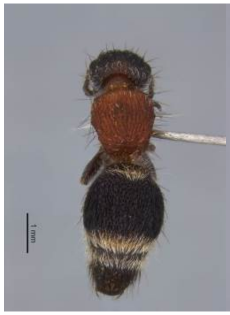
Dasylabroides ilythia

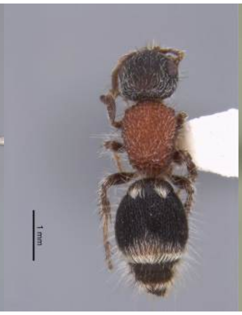
Dasylabroides latona schultzei

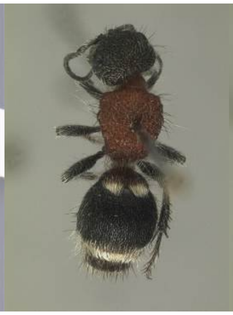
Dasylabroides katonga

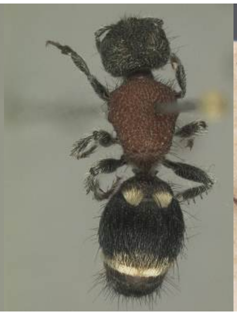
Dasylabroides neavei

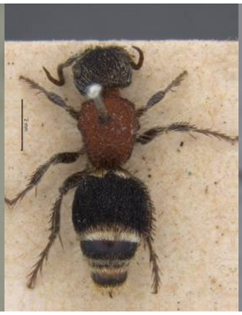
Dasylabroides nortia

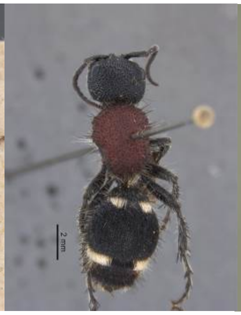
Dasylabroides cf. *neaveiformis*

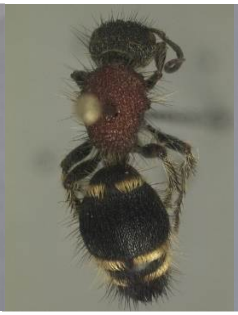
Dasylabroides sp.

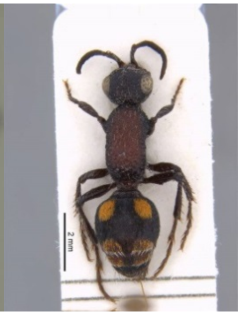
Dentotilla garuana

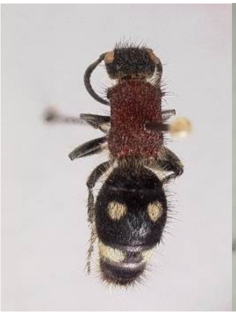
Diacanthotilla diacantha

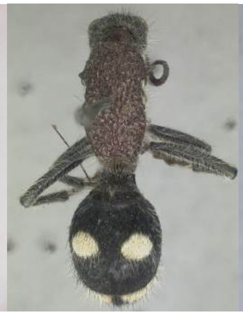
Dolichomutilla carsoni

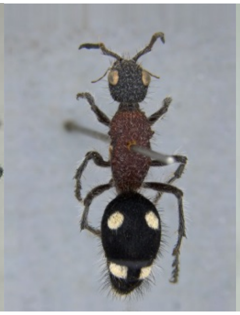
Dolichomutilla kibotonoensis

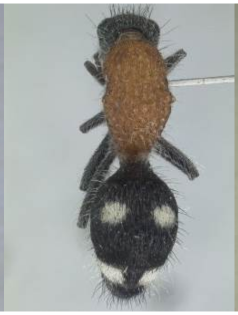
Dolichomutilla livingstonis

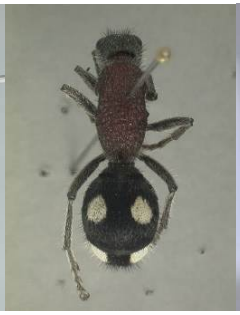
Dolichomutilla sycorax

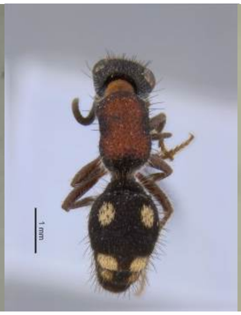
Glossotilla cassinensis

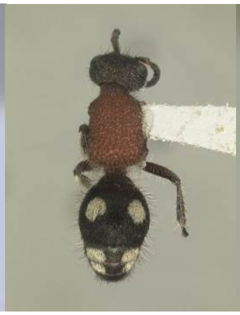
Glossotilla congoensis

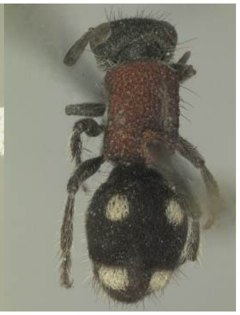
Glossotilla phacopus

COSMOPOLITAN RING

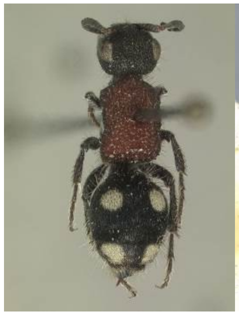

Glossotilla rukuruensis

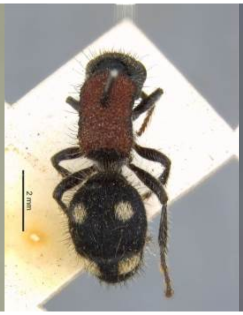

Glossotilla cf. casignete gandana

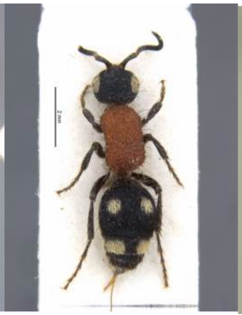

Glossotilla cf. mashonensis

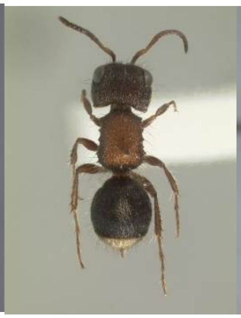

Labidomilla fuscipalis

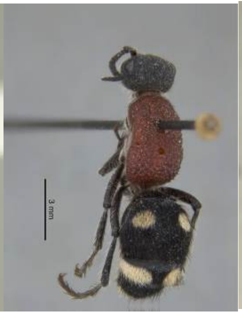

Lobotilla charaxus

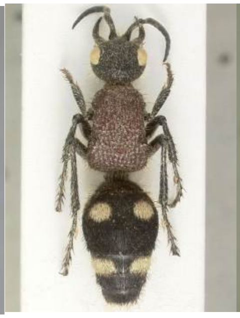

Lobotilla leucopyga

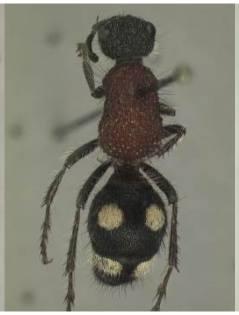

Lobotilla leucospila

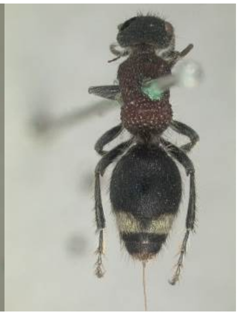

Mimecotilla glabrata

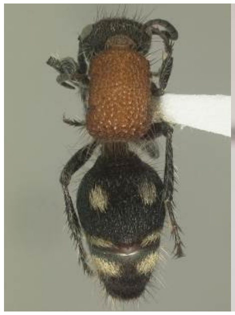

Mimecotilla granulopygidialis granulopygidialis

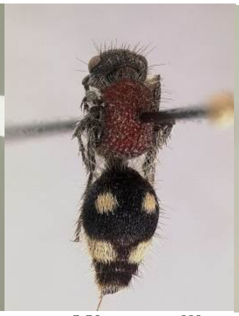

Mimecotilla granulopygidialis rugosipygialis

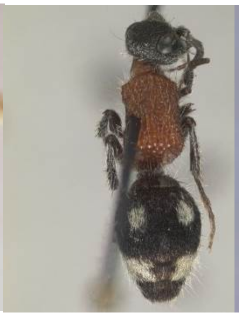

Mimecotilla landanensis

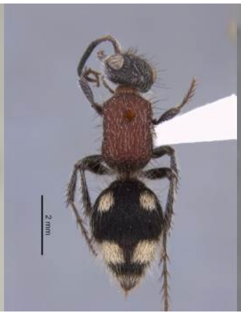

Mimecotilla vestita

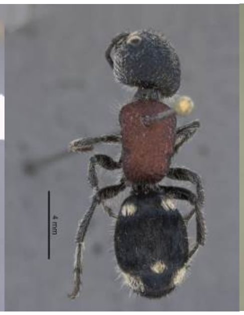

Mutilla astarte

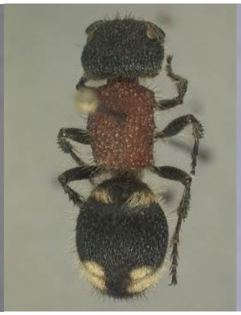

Mutilla diselena basiornata

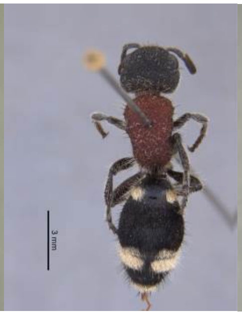

Mutilla diselena diselena

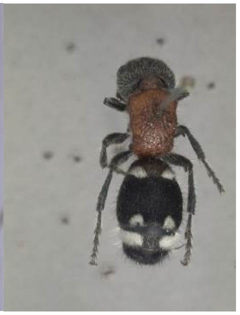

Mutilla diselena germanica

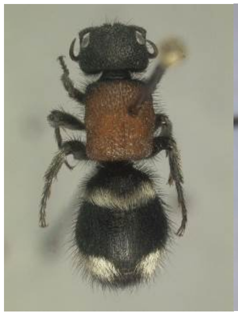

Mutilla europaea

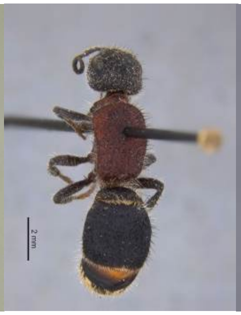

Mutilla incospicua

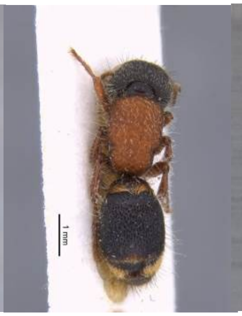

Mutilla nairobiana

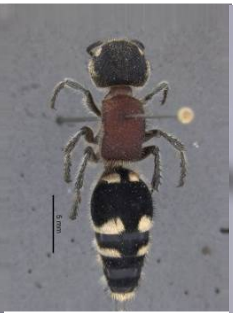

Mutilla parallela

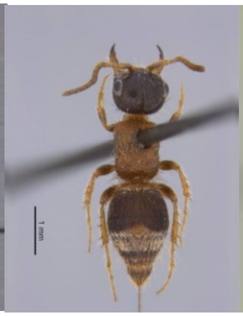

Myrmilla georgiae

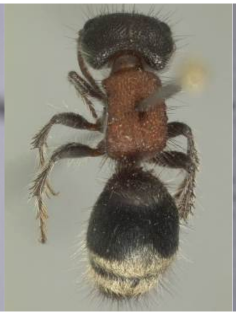

Myrmilla glabrata

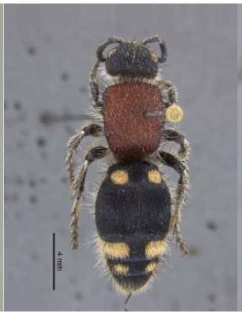

Odontomutilla calida calida

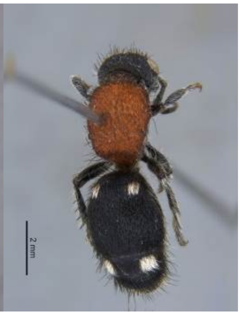

Odontomutilla fracta fracta

COSMOPOLITAN RING

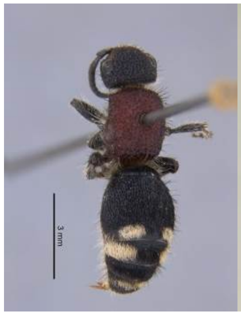
Odontomutilla fracta ocularis

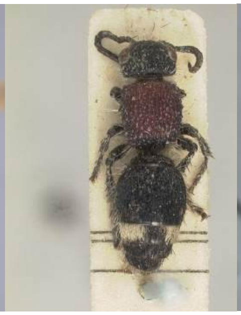
Odontomutilla fracta unifasciata

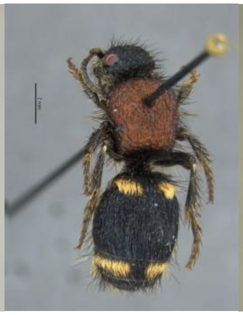
Odontomutilla notata notata

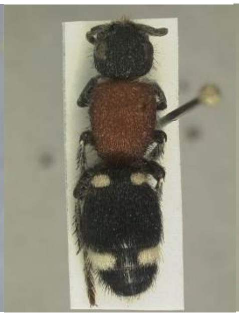
Odontomutilla notata umtalina

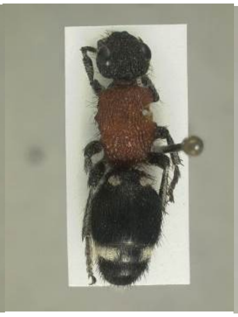
Odontomutilla zimrada kenyana

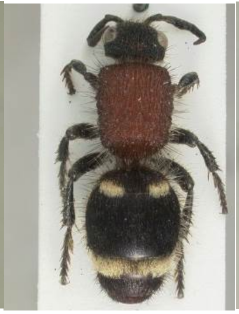
Odontomutilla aff. *mocquerysi*

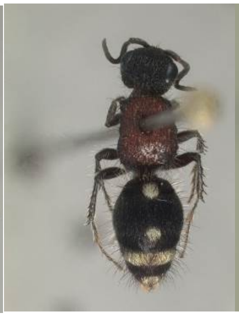
Odontotilla bidentata

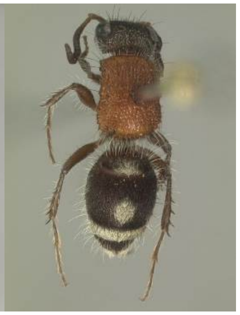
Odontotilla tridentata

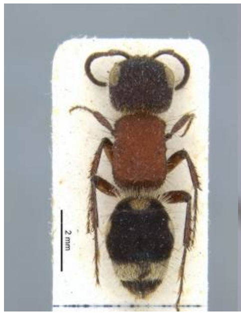
Odontotilloides trimaculata

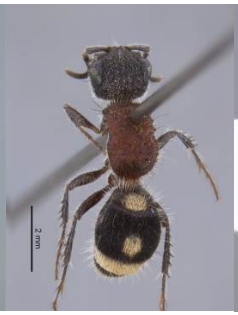
Odontotilloides dakarensis

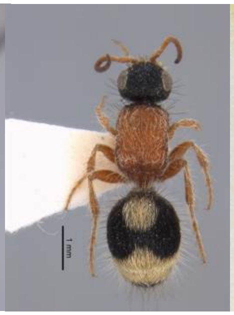
Physetopoda eltihnica

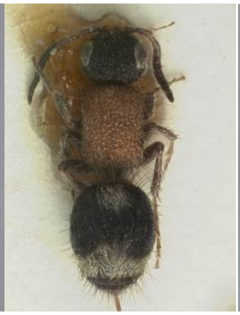
Physetopoda unicinta

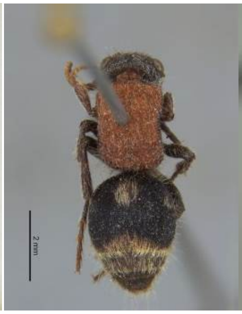
Physetopoda sp.

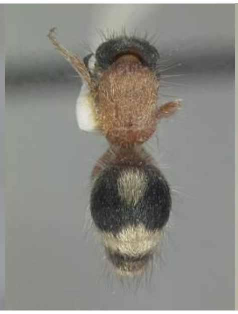
Physetopoda cf. *deserticola*

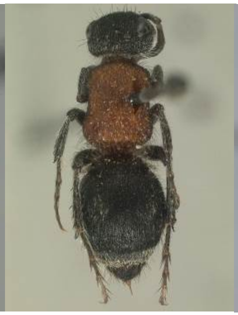
Pristomutilla clarior

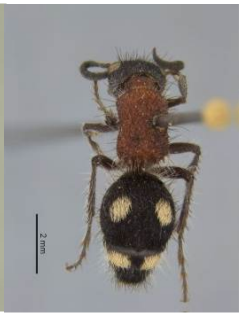
Pristomutilla congoana

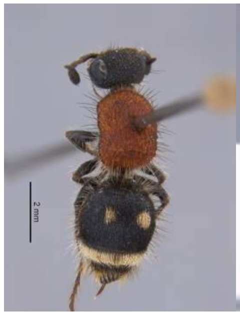
Pristomutilla ctenophora

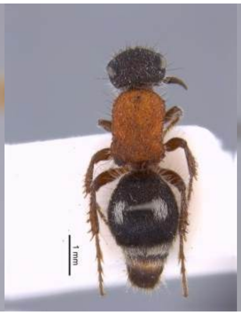
Pristomutilla ctenoterga

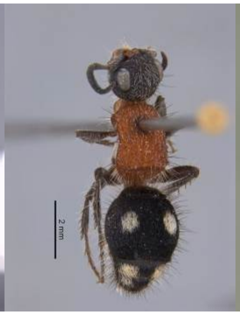
Pristomutilla dentidorsis

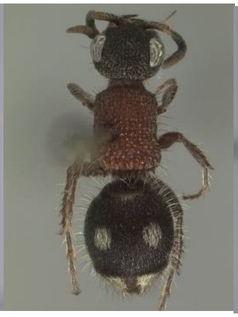
Pristomutilla heptaspila,

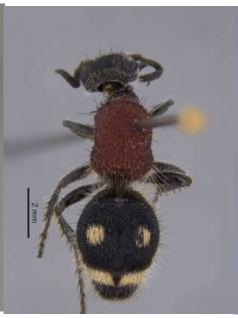
Pristomutilla maculata

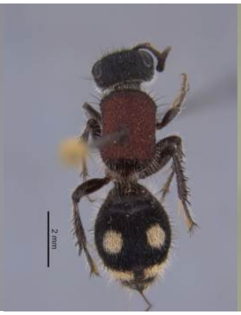
Pristomutilla multisignata

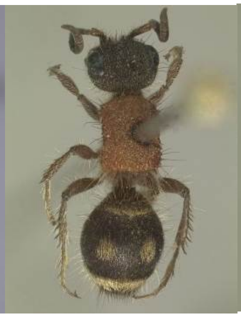
Pristomutilla rectistriata

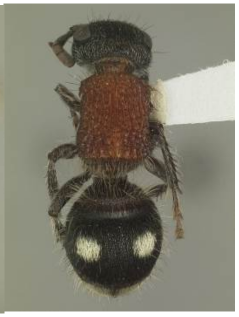
Pristomutilla sp.

COSMOPOLITAN RING

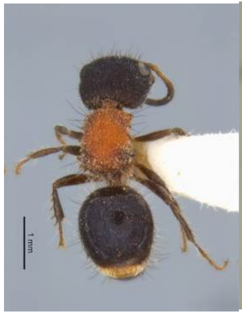
Pygomilla concava

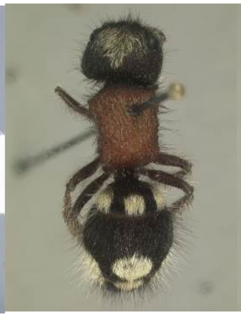
Ronisia barbara

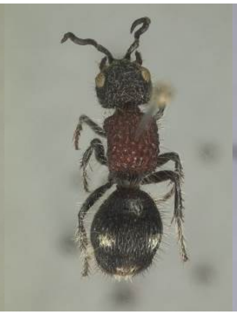
Ronisia penetrata agave

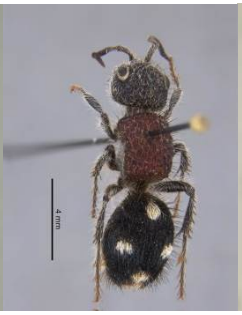
Ronisia penetrata wabonina

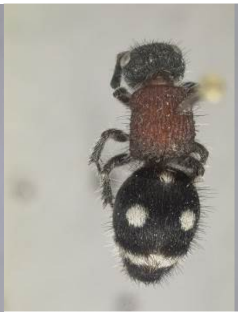
Ronisia trispilota straba

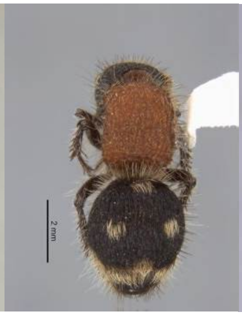
Ronisia trispilota trispilota

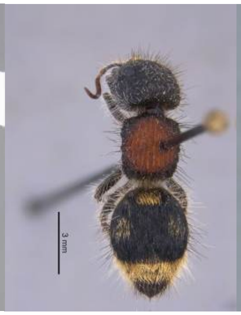
Ronisia zulu

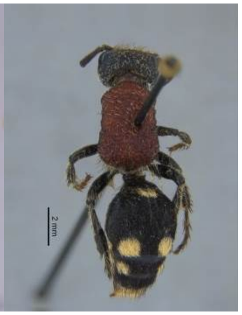
Seriatropsidia prastosemata

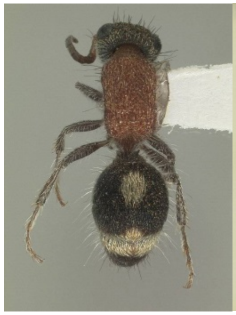
Smicromyrme difficilis

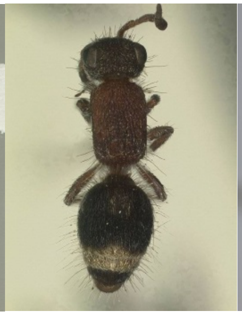
Smicromyrme dolichothoracica

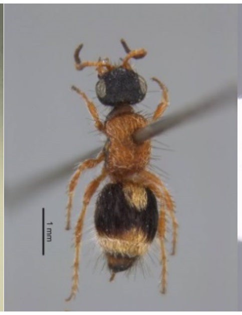
Smicromyrme gineri

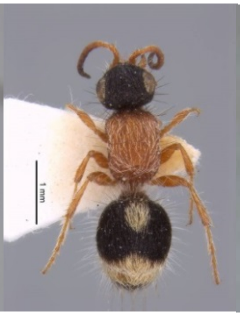
Smicromyrme lybica

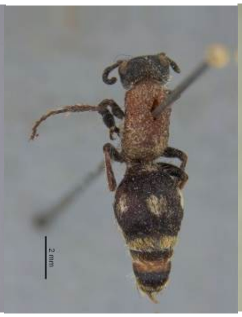
Smicromyrme tettensiella

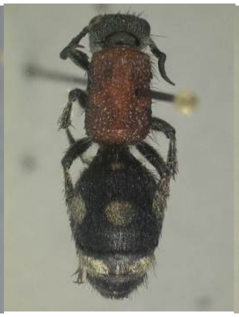
Smicromyrme tettensis quintociliata

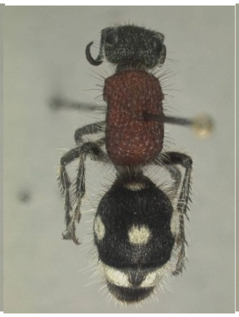
Smicromyrme tettensis tettensis

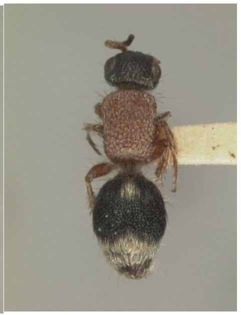
Smicromyrme cf. maidli

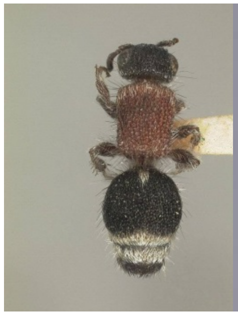
Smicromyrme cf. omphale

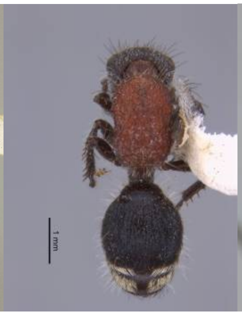
Spinulomutilla kifaruana

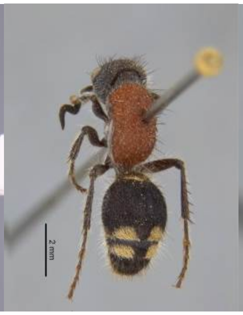
Spinulomutilla malaissei

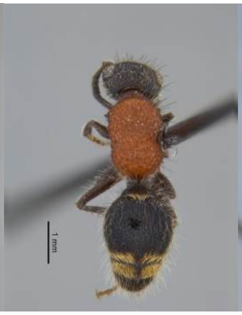
Spinulomutilla sp.

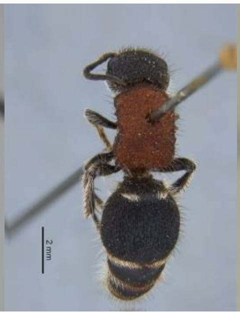
Spinulotilla sp.

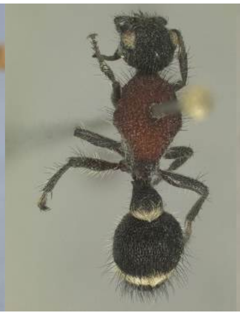
Stenomutilla albicaudata

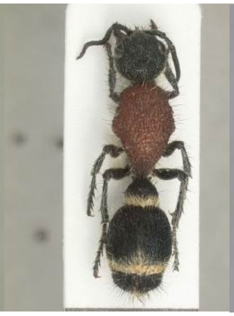
Stenomutilla analis

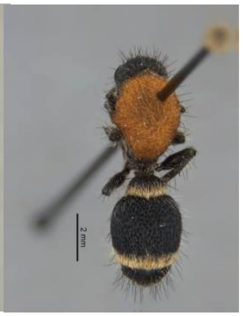
Stenomutilla baucis

COSMOPOLITAN RING

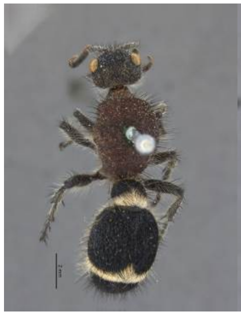
Stenomutilla beroe

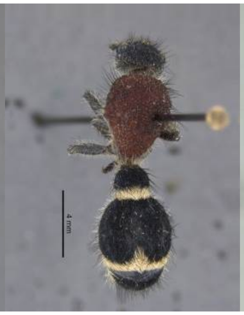
Stenomutilla bispina

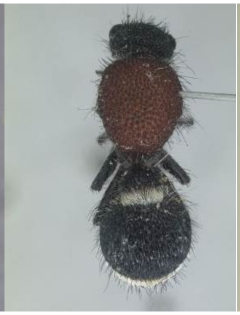
Stenomutilla curtithorax

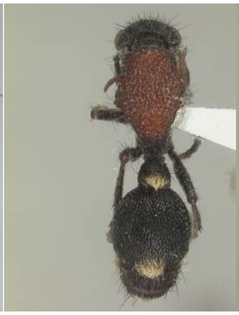
Stenomutilla dolichoderoides

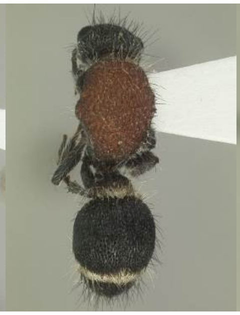
Stenomutilla pygidialis

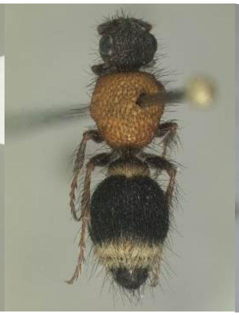
Stenomutilla sabulosa

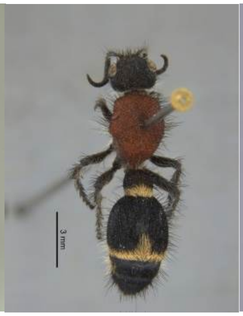
Stenomutilla schulthessi

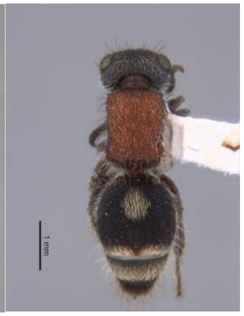
Strangulotilla minuta

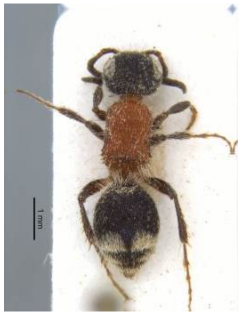
Strangulotilla samharica katanga

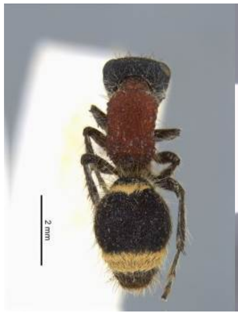
Strangulotilla silverlocki

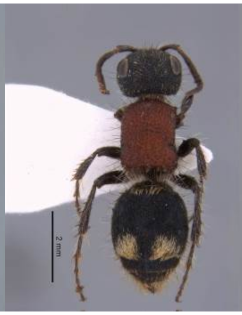
Strangulotilla strongylocerphala

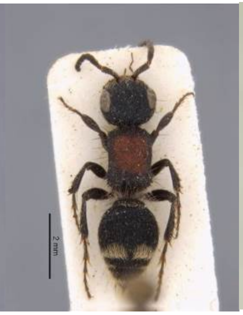
Strangulotilla thoracosulcata

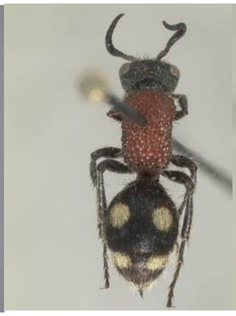
Trispilotilla liopyga

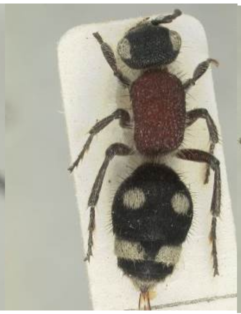
Trispilotilla telamon

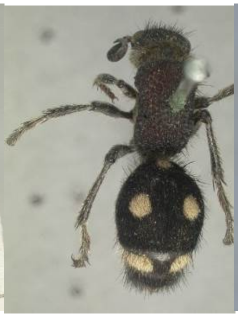
Trispilotilla trimaculata dewitziana

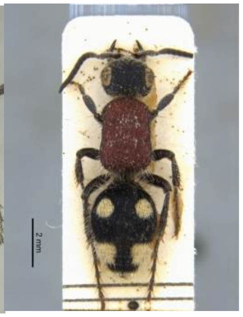
Trogaspidia aurata

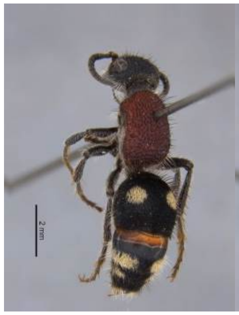
Trogaspidia bismarckburgensis

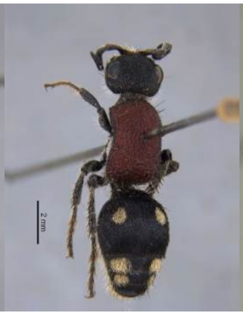
Trogaspidia buziana

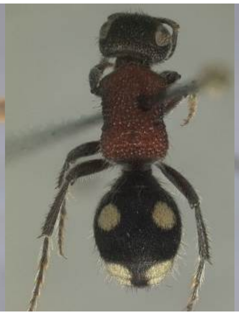
Trogaspidia chariensis

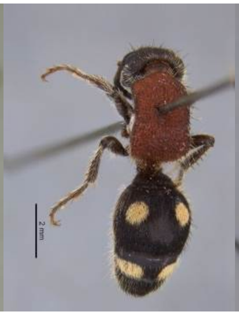
Trogaspidia ghindiana

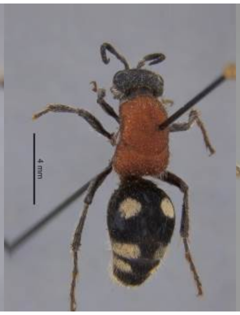
Trogaspidia guessfeldti

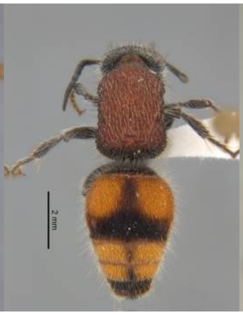
Trogaspidia heyderi

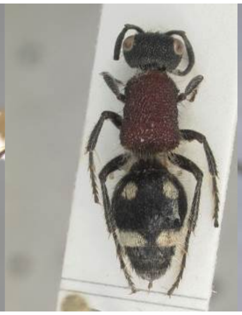
Trogaspidia intraorbitalis

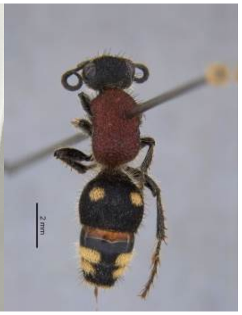
Trogaspidia keteana

COSMOPOLITAN RING

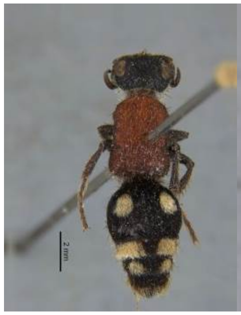

Trogaspidia* *kipochiana

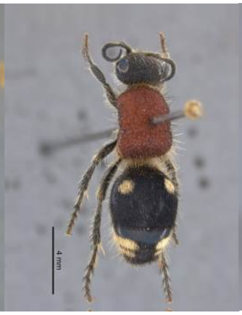

Trogaspidia* *major

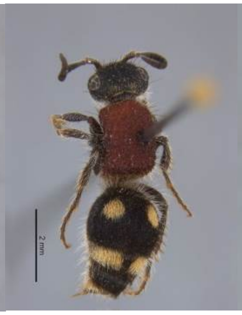

Trogaspidia* *pycnothoracica

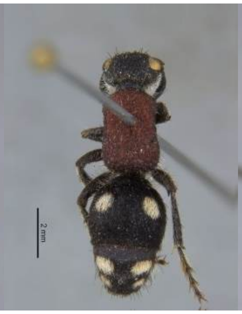

Trogaspidia* *reimeri

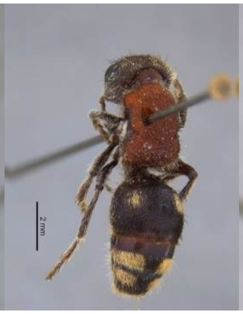

Trogaspidia* *rugulifera

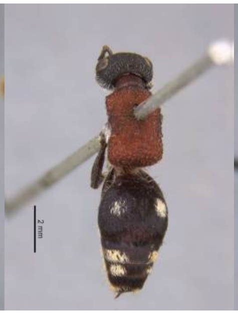

Trogaspidia* *salisburyensis

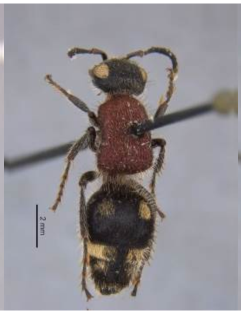

Trogaspidia* *sansibarensis

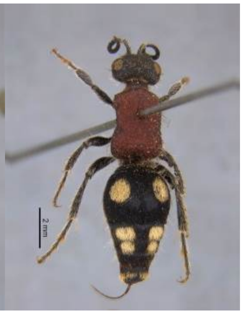

Trogaspidia* *sennarensis

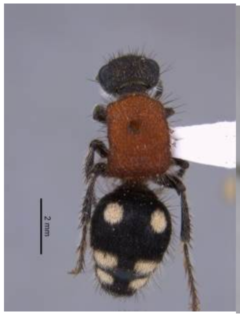

Trogaspidia* *sulcicada

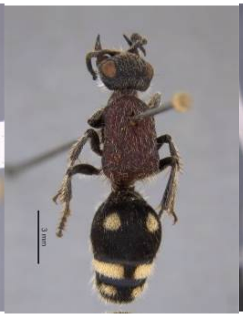

Trogaspidia* *tuberculifera

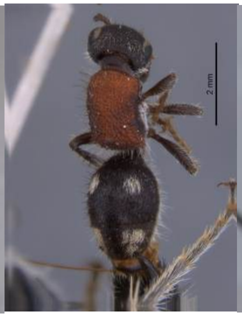

Trogaspidia* *uremana

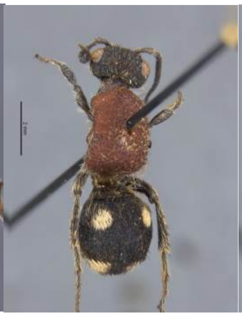

Trogaspidia **cf. *chikawana***

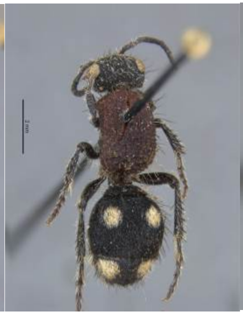

Trogaspidia **sp. nov.**

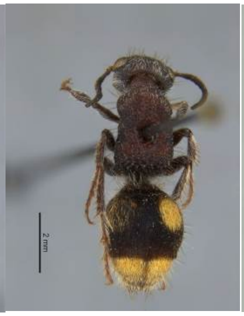

Vanhartenidia* *pyrrhospilota

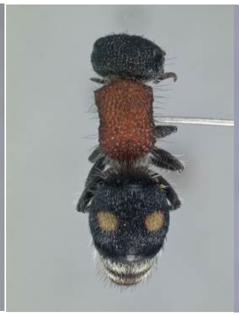

Viereckia* *acrisione

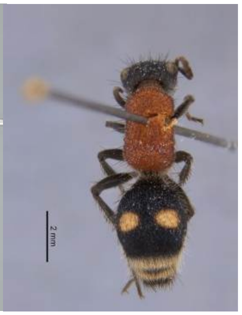

Viereckia* *bassutana

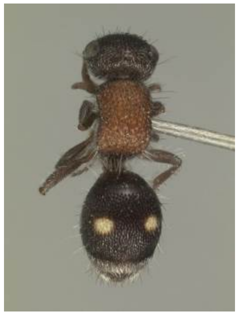

Viereckia **sp.**

Other Cosmopolitan Ring species not pictured:

Areotilla perplexa
Pseudocephalotilla beirana

EQUATORIAL RING

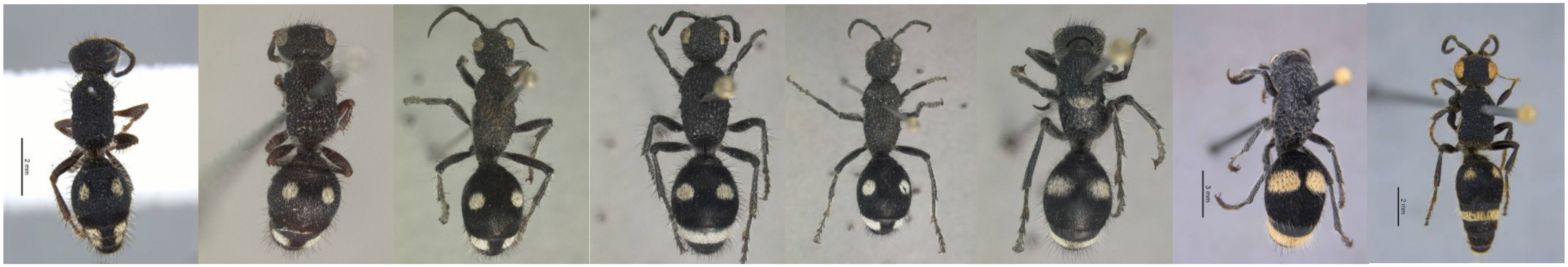

Carinotilla cf. *consors* | *Carinotilla* cf. *stilpnopyga* | *Dolichomutilla heterodonta* | *Dolichomutilla nigra fasciata* | *Dolichomutilla nigra nigra* | *Dolichomutilla scutellata* | *Dolichomutilla* cf. *andrei* | *Glossotilla atricolor*

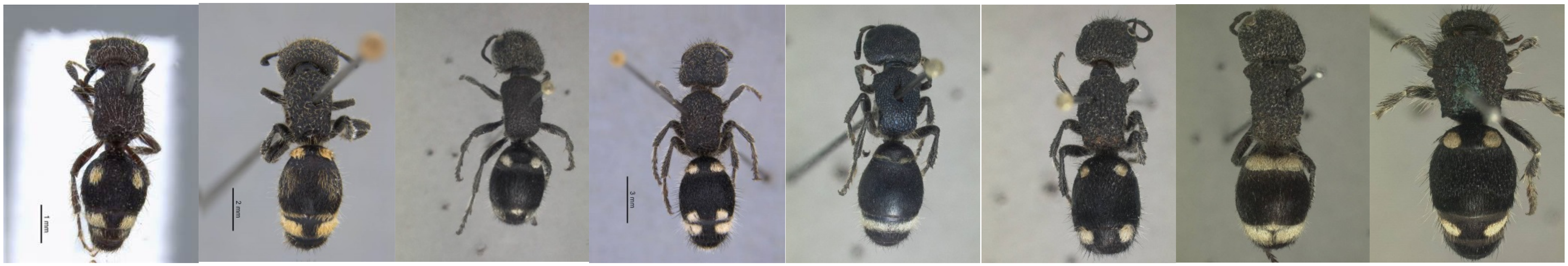

Glossotilla suavis speculatrix | *Mutilla alticola* | *Mutilla astarte ignava* | *Mutilla diselena obscurior* | *Mutilla porosicollis* | *Mutilla radamae* | *Mutilla scabrofoveolata kalaharica* | *Odontomutilla aegrota*

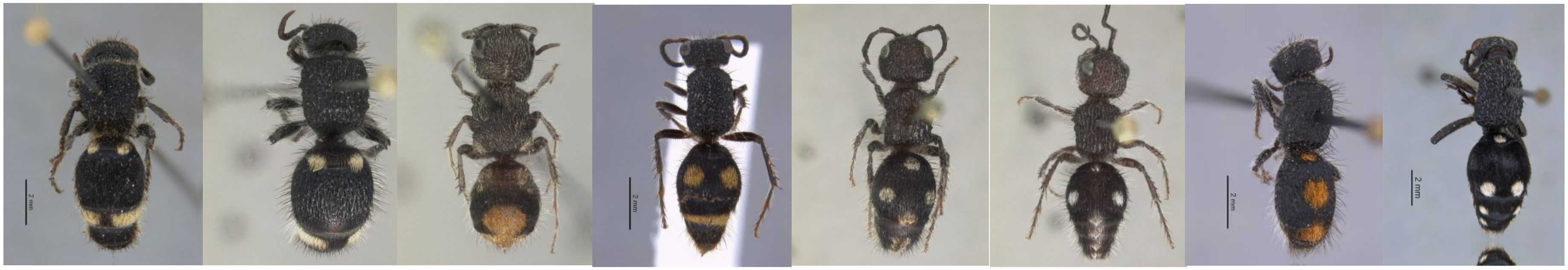

Odontomutilla mocquerysi | *Odontomutilla notata nigrita* | *Omotilla grazianii eugenii* | *Pristomutilla octacantha* | *Ronisia* cf. *gananina* | *Ronisia* cf. *meridioccidentalis* | *Ronisia* sp. nov. | *Seriatopsidia biseriata*

EQUATORIAL RING

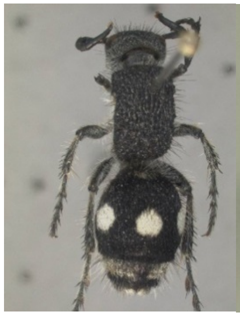

Smicromyrme tettensis melanothoracica

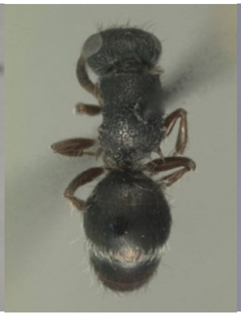

Smicromyrmilla sp.

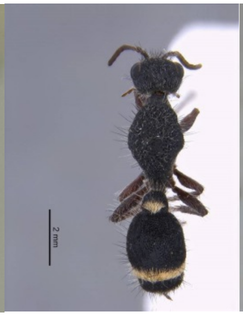

Stenomutilla kohli

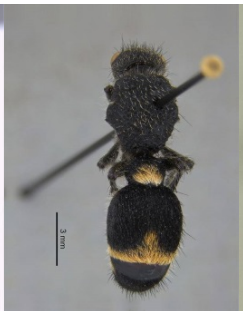

Stenomutilla mangocheana

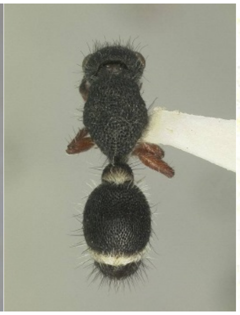

Stenomutilla rufipes

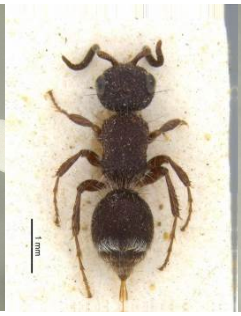

Strangulotilla thoracosulcata nigrithoracia

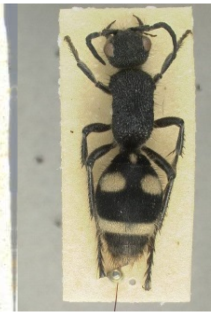

Trispilostilla africana

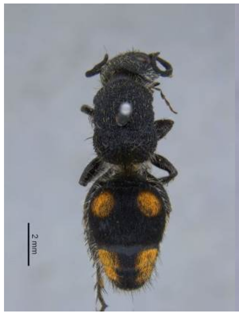

Trogaspidia somalica

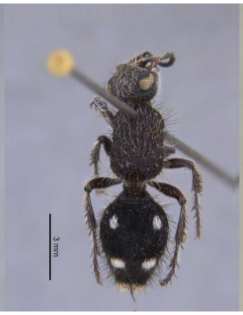

Trogaspidia syntoma

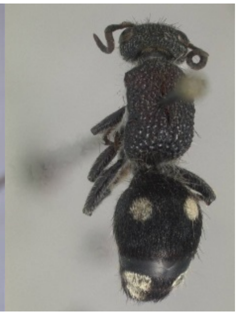

Trogaspidia cf. medon

MEDITERRANEAN-STEPPE RING

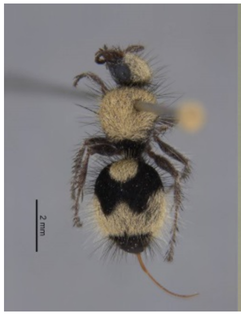

Dasylabris arabica

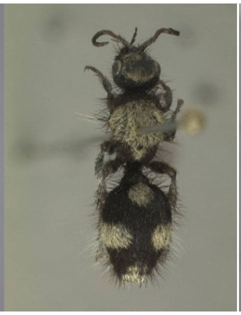

Dasylabris atrata

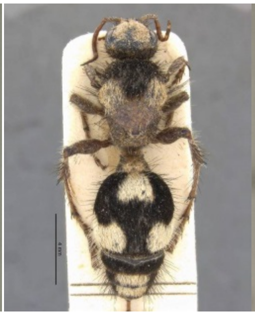

Dasylabris egregia

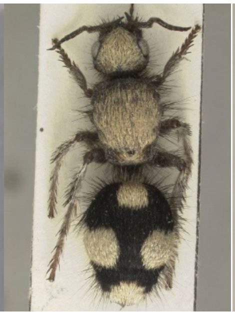

Dasylabris juxtarenaria

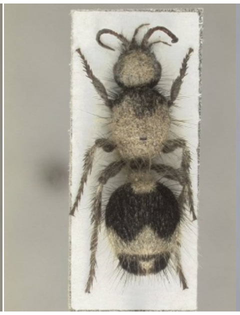

Dasylabris lugubris

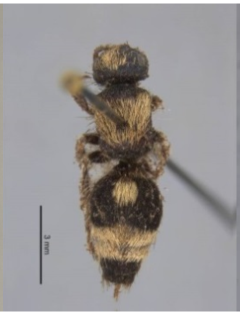

Nemka viduata tunensis

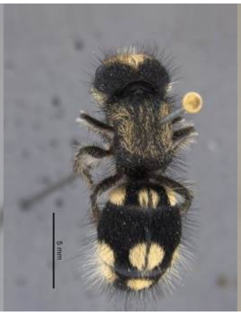

Ronisia maculosa

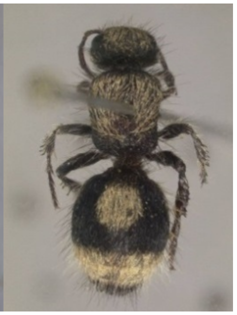

Smicromyrme mareotica

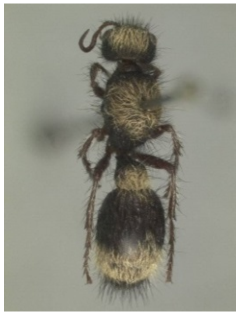

Stenomutilla argentata

Velvet ants that did not conform into one of the four mimicry rings

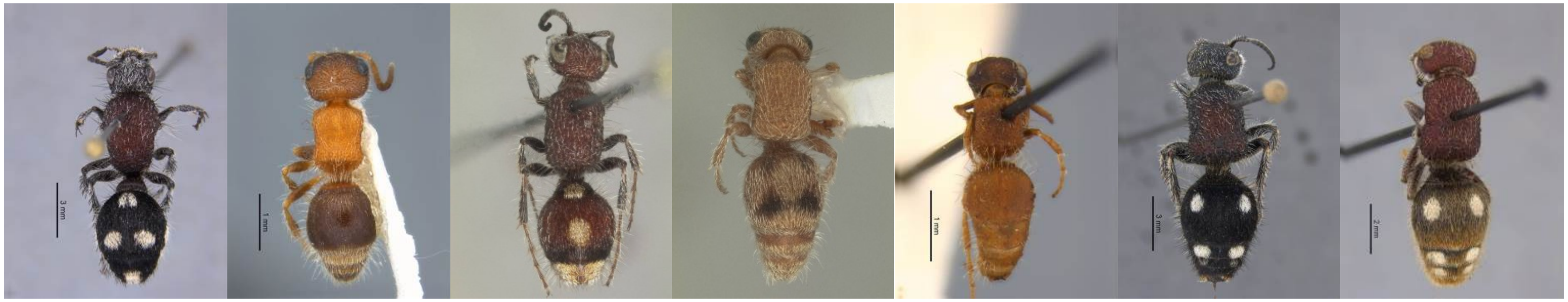

Dasylabris terpsichore | ***Montanomutilla*** **cf.** ***bella*** | ***Smicromyrme carosellii*** | ***Smicromyrme rufescens*** | ***Stranuglotilla parva*** | ***Trogaspidia luangwicola*** | ***Trogaspidia mombasana***

Other non-mimicry ring conforming species not pictured:

Rhopalomutilla carinaticeps
